# Clumping factor B is an important virulence factor during *Staphylococcus aureus* skin infection and a promising vaccine target

**DOI:** 10.1371/journal.ppat.1007713

**Published:** 2019-04-22

**Authors:** Keenan A. Lacey, Michelle E. Mulcahy, Aisling M. Towell, Joan A. Geoghegan, Rachel M. McLoughlin

**Affiliations:** 1 Host-Pathogen Interactions Group, School of Biochemistry and Immunology, Trinity Biomedical Sciences Institute, Trinity College Dublin, Dublin, Ireland; 2 Department of Microbiology, Moyne Institute of Preventive Medicine, School of Genetics and Microbiology, Trinity College Dublin, Dublin, Ireland; Columbia University, UNITED STATES

## Abstract

*Staphylococcus aureus* expresses a number of cell wall-anchored proteins that mediate adhesion and invasion of host cells and tissues and promote immune evasion, consequently contributing to the virulence of this organism. The cell wall-anchored protein clumping factor B (ClfB) has previously been shown to facilitate *S*. *aureus* nasal colonization through high affinity interactions with the cornified envelope in the anterior nares. However, the role of ClfB during skin and soft tissue infection (SSTI) has never been investigated. This study reveals a novel role for ClfB during SSTIs. ClfB is crucial in determining the abscess structure and bacterial burden early in infection and this is dependent upon a specific interaction with the ligand loricrin which is expressed within the abscess tissue. Targeting ClfB using a model vaccine that induced both protective humoral and cellular responses, leads to protection during *S*. *aureus* skin infection. This study therefore identifies ClfB as an important antigen for future SSTI vaccines.

## Author summary

*Staphylococcus aureus* is the leading cause of skin and soft tissue infections (SSTIs), the treatment of which is becoming increasingly difficult due to antibiotic resistance. An anti-*S*. *aureus* vaccine offers a potential solution, but a better understanding of how *S*. *aureus* causes pathology during SSTI is required to identify effective vaccine targets. Here, we identify an important virulence determinant during *S*. *aureus* SSTI. Clumping factor B (ClfB), a surface protein expressed by *S*. *aureu*s is shown to promote skin abscess formation by binding to the host protein loricrin. Targeting ClfB using a model vaccine conferred significant protection during *S*. *aureus* SSTI. In this study, we uncover an entirely novel mechanism by which *S*. *aureus* forms abscesses during skin infection, identifying an important therapeutic target for treating *S*. *aureus* SSTI.

## Introduction

*Staphylococcus aureus* is the leading cause of skin and soft tissue infections (SSTIs) in humans [1, 2] with *S*. *aureus* SSTIs resulting in over 11 million outpatient visits and almost 500,000 hospital admissions in the United States annually [3]. *S*. *aureus* SSTIs routinely manifest as cutaneous abscesses which limit the penetration and efficacy of antibiotics [4]. Community-acquired methicillin resistant *S*. *aureus* (CA-MRSA) skin infections are increasing in frequency in healthy individuals [5] and the treatment of these infections has become increasingly difficult due to the emergence of antibiotic resistance. The development of an anti-*S*. *aureus* vaccine offers a potential solution to prevent infection regardless of antibiotic resistance; however, although significant efforts have been made, an effective anti-*S*. *aureus* vaccine remains elusive.

Given the wide spectrum of pathologies caused by this bacterium it is unlikely that a universal anti-*S*. *aureus* vaccine will ever be realised [6], and instead, a vaccine targeting specific clinical manifestations may need to be pursued. As skin is the most frequent site of *S*. *aureus* infection [7], a vaccine specifically targeted against SSTIs would be of great benefit. If prophylactic vaccines or other forms of immunotherapy to treat *S*. *aureus* SSTIs are to be developed as an alternative to antibiotics, a greater understanding of the specific role of individual virulence factors during infection at this site is needed to identify important targets for future therapy.

*S*. *aureus* expresses up to 25 different cell wall-anchored (CWA) proteins, which are covalently linked to the peptidoglycan layer by the enzyme sortase A and are primarily involved in adhesion and invasion of host cells and tissues, biofilm formation and immune evasion [8]. Sortase A-deficient mutants, which lack the majority of cell surface bound CWA proteins, have reduced virulence in a murine kidney abscess model [9, 10], while in a skin abscess model, a sortase A-deficient mutant resulted in lower bacterial burden in the skin and a significantly reduced pathology compared to wild-type infected mice [11]. These studies indicate that CWA proteins are important during SSTIs and suggest that CWA proteins may be particularly important during the process of abscess formation. However, there is a paucity of information regarding the role of individual surface proteins during SSTIs. Clumping factor A has previously been shown to play a role in the pathogenesis of *S*. *aureus* SSTI, as mice inoculated with a ClfA-deficient mutant of *S*. *aureus* strain Newman demonstrated a lower bacterial burden in the skin compared to the wild type strain at Day 2 post-inoculation [11]. However, vaccination strategies targeting ClfA only provided modest protection in this model [12] suggesting that other surface proteins may also play an important role in the development of *S*. *aureus* SSTI.

*S*. *aureus* attachment to the anterior nares during colonization is facilitated by the CWA protein clumping factor B (ClfB) through high affinity interactions with the cornified envelope. Through this interaction ClfB has been shown to promote nasal colonization in both rodents and humans [13–15]. ClfB is expressed in the early exponential phase of growth and is absent from cells in the late and stationary phase. The N-terminus of ClfB consists of a signal sequence followed by its binding domain, region A. This is a 540 amino-acid long segment containing 3 independently folded subdomains, N1, N2 and N3 [16]. Similar to ClfA, the ligand-binding region of ClfB has been localised to the N2N3 domain of region A. The N1 domain has no known binding function [16]. The *clfB* gene is carried by most strains of *S*. *aureus* [17]. Sequence variation in the ClfB protein occurs between different clonal complexes of *S*. *aureus*, with the variant proteins sharing at least 94% amino-acid identity to each other [18]. ClfB binds to plasma fibrinogen [19], cytokeratin 10 [20], which is the dominant component of the interior of squamous cells, and to loricrin, which is the most abundant protein of the cornified envelope of squamous cells [21]. Rates of *S*. *aureus* nasal colonization were significantly reduced in loricrin-knockout mice compared to wild-type mice, demonstrating that loricrin is a critical ligand for ClfB *in vivo*, at least in mice [13]. The contribution of ClfB to *S*. *aureus* skin infection however, has never previously been addressed. This study aims to elucidate its role during *S*. *aureus* skin infection and to assess its potential as a candidate vaccine antigen specifically targeting SSTIs.

## Results

### ClfB is an important virulence factor during *S*. *aureus* SSTI

**Fig 1:**
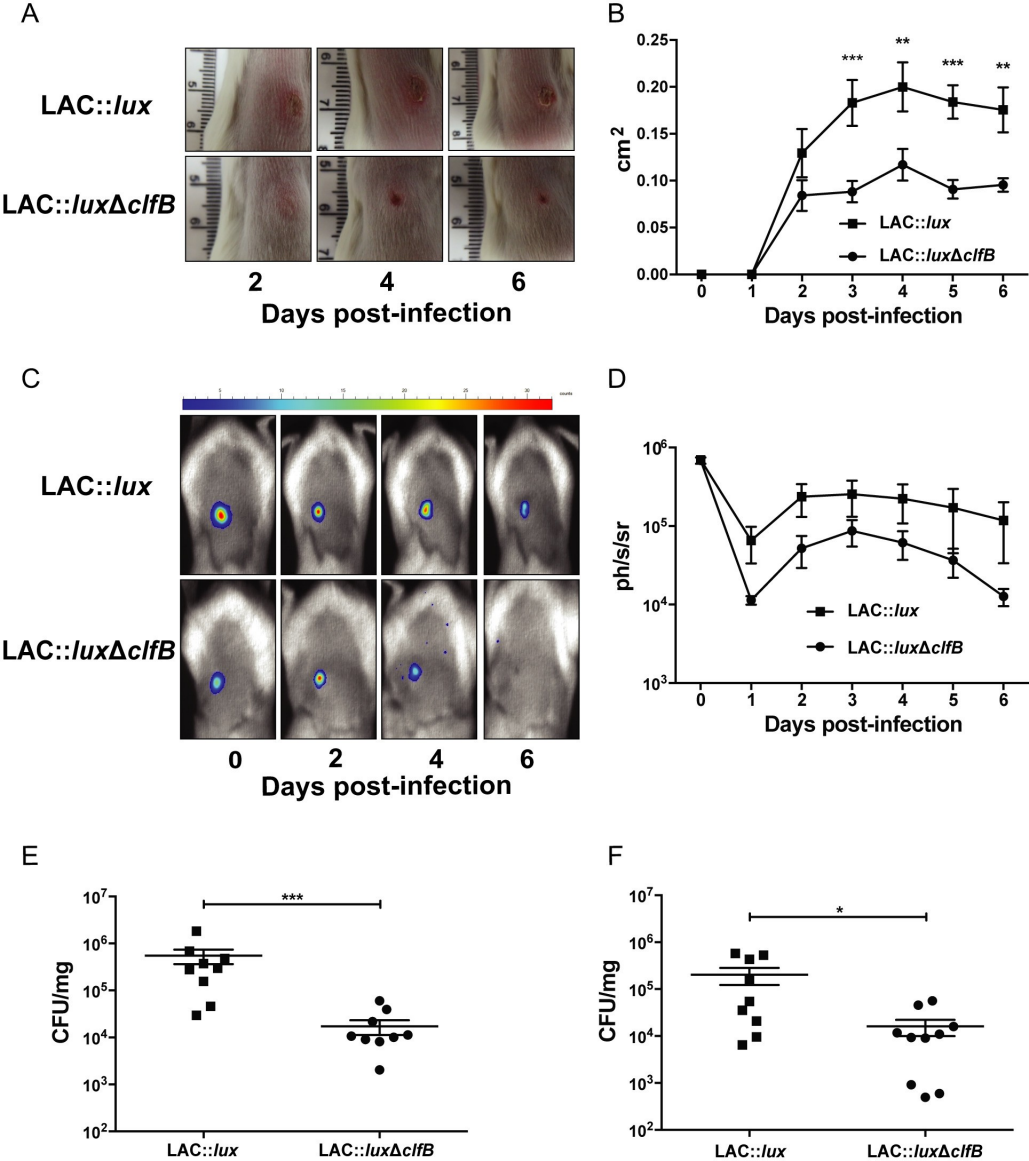
ClfB contributes to virulence during *S. aureus* SSTI. BALB/c mice were infected subcutaneously with 2x10^7^ CFU *S. aureus* LAC::*lux* or LAC::*lux* Δcl*fB* and abscess lesion size and bacterial burden was measured. Representative lesions from the dorsal area of mice from each group are shown (A) and results are expressed as total lesion size (cm^2^) ± SEM (B). Bioluminescence imaging was carried out using a Photon Imager and representative *in vivo* bioluminescence images are shown (C). Results are expressed as mean total photon flux (photons per second per steradian) ± SEM (D). Bacterial burden in the skin was assessed by viable counting on day 3 (E) and day 6 (F) post-infection. Results are expressed as Log10 CFU/mg. n = 9–10 per group. Data pooled from 2 independent experiments. Two-way ANOVA with Bonferroni post-test (B) and Mann-Whitney U test (E, F) used to analyse differences between groups. * P < 0.05, ** P < 0.01, *** P < 0.001.

To evaluate the role of ClfB during *S*. *aureus* SSTIs, groups of BALB/c mice were inoculated subcutaneously (s.c.) with 2x10^7^ CFU *S*. *aureus* LAC::*lux* or LAC::*lux* Δ*clfB*. Abscess lesion area was measured and bioluminescence imaging of mice was recorded over a six-day infection period. Mice infected with LAC::*lux* Δ*clfB* formed significantly smaller abscess lesions on day 3–6 compared to mice infected with LAC::*lux* (Fig 1A and 1B). The bioluminescence signal was also reduced in LAC::*lux* Δ*clfB* infected animals compared to LAC::*lux* infected mice (Fig 1C and 1D). The reduction in bioluminescence signal was validated by quantifying the bacterial burden in the skin on day 3 (Fig 1E) and day 6 (Fig 1F). Importantly there was no difference in initial bacterial burden in the skin at 6 hours post challenge between LAC::*lux* Δ*clfB* and LAC::*lux* infected mice (S1 Fig). There was some dissemination of *S*. *aureus* to systemic sites at 24 hours post challenge but this was not different between LAC::*lux* Δ*clfB* infected animals and LAC::*lux* infected mice (S1 Table). By day 3 post challenge with LAC::*lux* bacterial burden in the systemic organs was ≤ 1 Log CFU.

**Fig 2:**
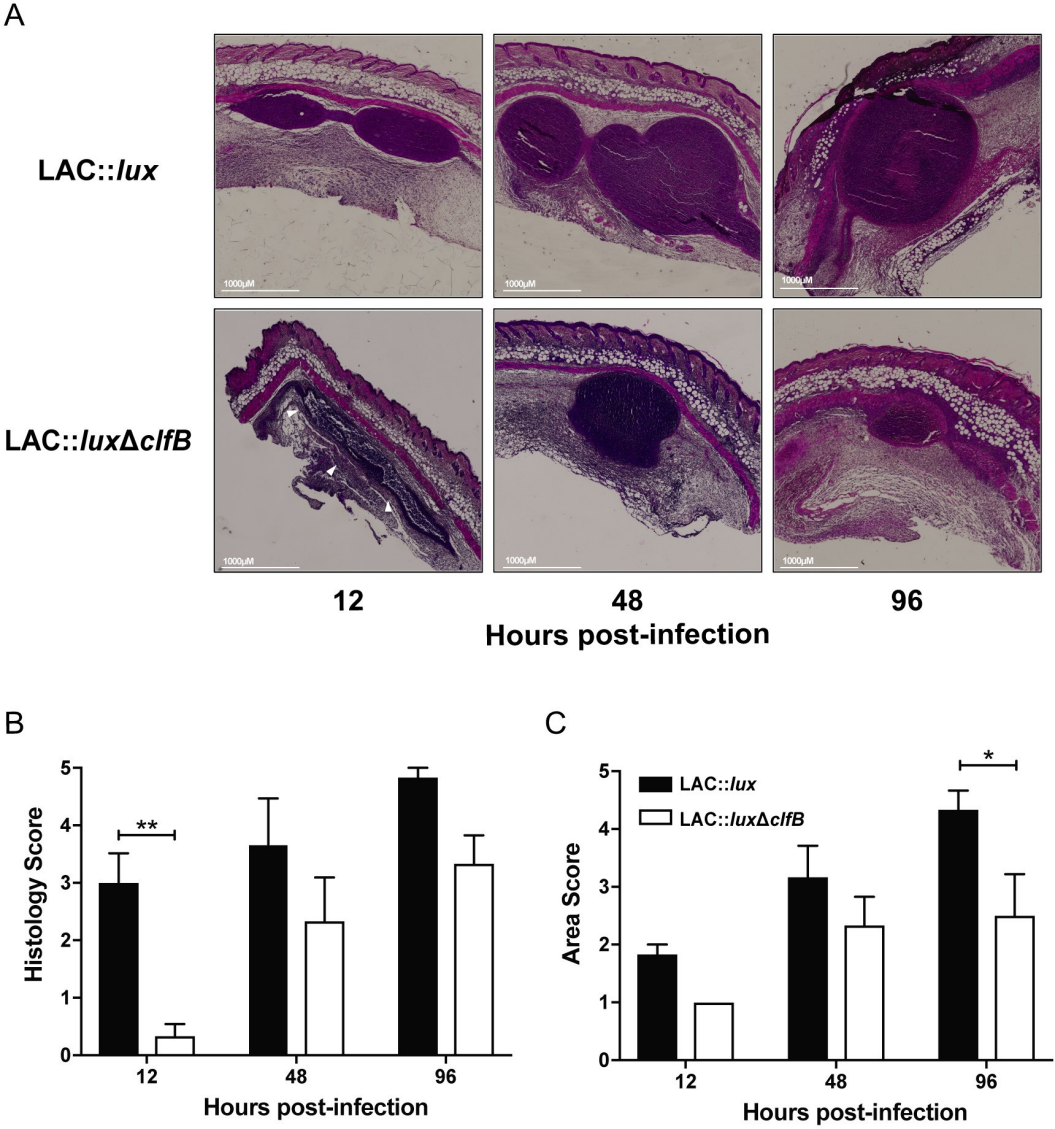
ClfB contributes to abscess structure and size during *S. aureus* SSTI. BALB/c mice were infected subcutaneously with 2x10^7^ CFU *S. aureus* LAC::*lux* or LAC::*lux* Δcl*fB* and abscess tissue was excised at 12, 48 and 96 hours. Tissue was fixed, embedded in paraffin wax and sectioned before haematoxylin and eosin staining was performed. Representative sections from each group are shown (A). White arrows indicate the lack of defined abscess wall structure. Tissue sections were scored (double blind) for histology score (B). Abscess area was computed using ImageJ software and the areas were scored accordingly (C). Results expressed as mean score ± SEM. n = 6 per group. Data pooled from 2 independent experiments. Two-way ANOVA with Bonferroni post-test used to analyze differences between groups (B, C). * P < 0.05, ** P < 0.01.

As the abscess lesion area was altered in LAC::*lux* Δ*clfB*-infected mice as early as 2 days post infection (Fig 1B), the role of ClfB during skin abscess formation was investigated by analysing skin excised at various time points throughout the first 96 hours of infection with either LAC::*lux* or LAC::*lux* Δ*clfB*. Haematoxylin and eosin staining was performed on sections to examine differences in abscess structure (Fig 2A). The abscesses from LAC::*lux* Δ*clfB*-infected animals were structurally distinct compared to those of LAC::*lux*-infected mice at 12 h post-infection, exhibiting bacteria spread throughout the skin and not encased within an abscess wall structure. Abscess structure score was significantly lower at 12 hours post-infection (Fig 2B), suggesting that ClfB may be important during the early stages of abscess formation. In addition, the overall abscess area remained reduced in LAC::*lux* Δ*clfB*-infected mice compared to LAC::*lux*-infected mice up to 96 h post-infection (Fig 2C).

Taken together, these results demonstrate that ClfB affects both the rate of abscess formation and the overall size of the abscess formed, which ultimately leads to greater bacterial burden in the skin. Abscess formation has been previously shown to facilitate *S*. *aureus* persistence within tissues [9]. Crucially, these results have demonstrated for the first time that ClfB has an important function during *S*. *aureus* SSTIs, likely exerting its effect early in the infection process.

### The ClfB-loricrin interaction is important for virulence during *S*. *aureus* SSTI

**Fig 3:**
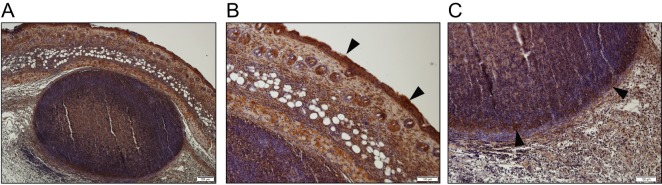
Loricrin in present within the skin abscess tissue of LAC::*lux* infected mice. BALB/c mice were infected subcutaneously with 2x10^7^ CFU *S. aureus* LAC::*lux* and abscess tissue was excised at 48h post-infection. Tissue was fixed, embedded in paraffin wax and sectioned before anti-loricrin staining (dark brown) was carried out. Low power field of the entire abscess structure is shown (A). Relevant tissue sections are shown in high power fields: skin epidermal layer (B) and abscess wall (C). Black arrows indicate the presence of loricrin. Representative images of n = 3 stained sections.

**Fig 4:**
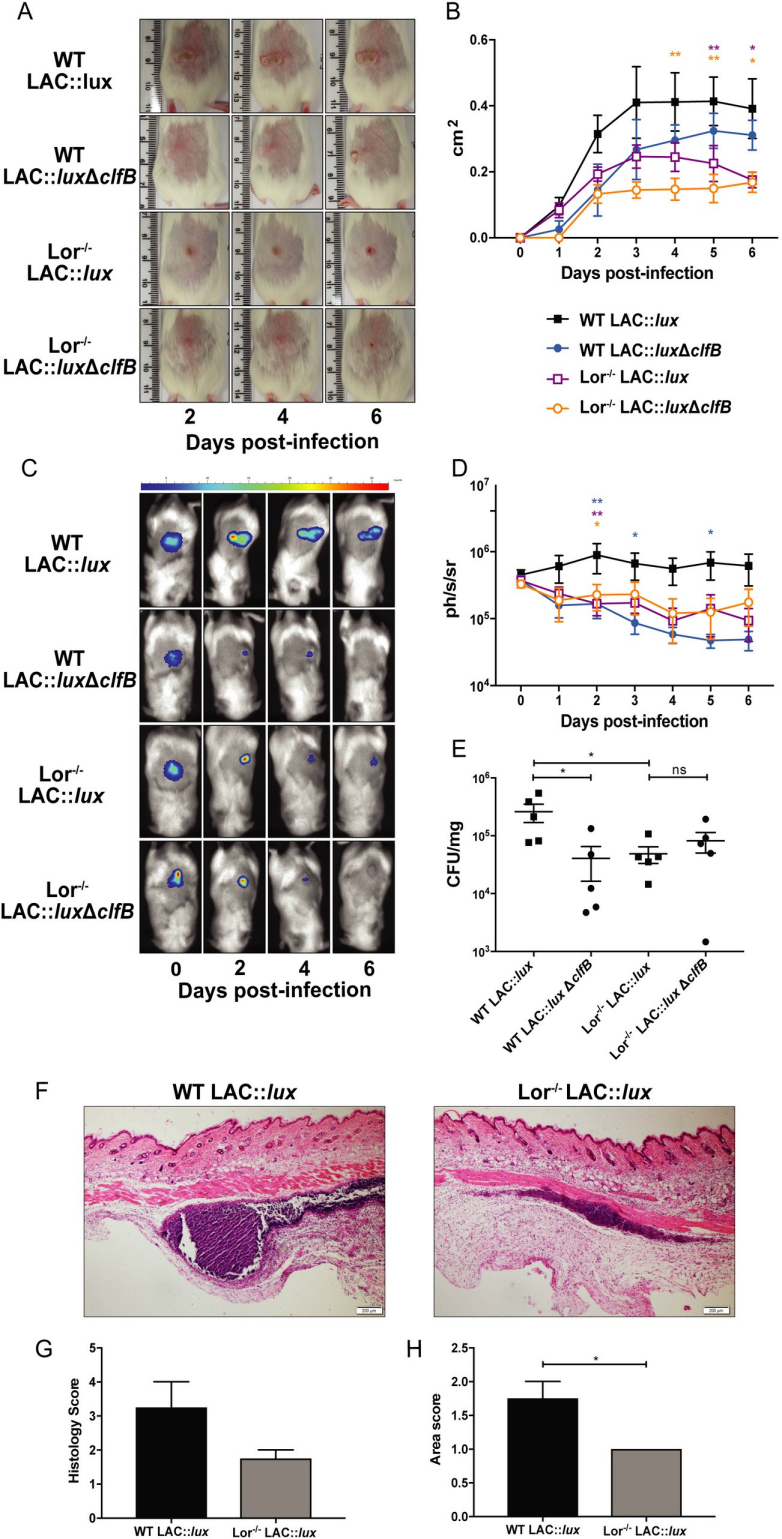
Loricrin is an important ligand during *S. aureus* SSTI. Wild-type FVB (WT) and Lor^-/-^ mice were infected subcutaneously with 2x10^7^ CFU *S. aureus* LAC::*lux* or LAC::*lux* Δcl*fB* and abscess lesion size and bacterial burden was measured. Representative lesions from the dorsal area of mice from each group are shown (A) and results are expressed as total lesion size (cm^2^) ± SEM (B). Bioluminescence imaging was carried out using a Photon Imager and representative *in vivo* bioluminescence images are shown (C). Results are expressed as mean total photon flux (photons per second per steradian) ± SEM (D). Bacterial burden in the skin was assessed by viable counting on day 6 (E) post-infection. Results are expressed as Log10 CFU/mg. n = 5 per group. Tissue sections from WT and Lor^-/-^ infected mice 12 h post-infection were stained with haematoxylin and eosin (F). Representative sections from each group are shown. Tissue sections were scored (double blind) for histology score (G). Abscess area was computed using ImageJ software and the areas were scored accordingly (H). Results expressed as mean score ± SEM. n = 4 per group. Two-way ANOVA with Bonferroni post-test (B, D), one-way ANOVA with Tukey post-test (E) and Mann-Whitney U test (G, H) used to analyze differences between groups. * P < 0.05, ** P < 0.01.

During nasal colonization, *S*. *aureus* binding to the squamous epithelium of the anterior nares is facilitated via the interaction between ClfB and the squamous cell envelope protein loricrin [13]. To investigate if the binding of loricrin by ClfB is important during SSTIs, the localisation of loricrin was investigated within the tissue during *S*. *aureus* subcutaneous abscess formation. At 48 h post-infection (Fig 3) loricrin is clearly visible in the epidermal layer of the skin (Fig 3B), which is to be expected as it is a major component of the cornified envelope. Interestingly, it is also detected in the abscess wall structure following challenge with both LAC::*lux* (Fig 3C) and LAC::*lux* Δ*clfB* (S2 Fig). To confirm the importance of the ClfB-loricrin interaction during SSTIs, wild-type FVB (WT) and loricrin-deficient mice (Lor^-/-^) were infected s.c. with LAC::*lux* and abscess lesion area and bioluminescence quantified. Lor^-/-^ mice formed significantly smaller abscesses (Fig 4A and 4B) compared to WT mice and had reduced bioluminescence (Fig 4C and 4D), indicating the presence of loricrin is important for abscess formation and bacterial burden during *S*. *aureus* SSTIs. Importantly, it can be seen that abscess formation is impaired in Lor^-/-^ mice at 12 hours post infection (Fig 4F) with overall less well-structured (Fig 4G) and smaller (Fig 4H) abscesses being formed. To investigate if a ClfB-loricrin specific interaction was required for virulence in this model, WT and Lor^-/-^ mice were also infected with LAC::*lux* Δ*clfB*. As expected, WT mice that received LAC::*lux* Δ*clfB* formed smaller abscess lesions (Fig 4A and 4B) and had significantly reduced bioluminescence (Fig 4C and 4D) compared to WT mice that received LAC::*lux* which was confirmed by quantifying bacterial burden in the skin (Fig 4E). In contrast, there was no significant difference in abscess lesion area (Fig 4A and 4B), bioluminescence (Fig 4C and 4D) or skin CFUs (Fig 4E) between Lor^-/-^ mice that received LAC::*lux* or LAC::*lux* Δ*clfB*. Furthermore there was no difference in the overall size or structure of the abscess formed in Lor^-/-^ mice that received LAC::*lux* or LAC::*lux* Δ*clfB* (S3 Fig). Taken together these data suggest that expression of loricrin within the tissue during abscess formation facilitates the binding of *S*. *aureus* specifically via ClfB, thus contributing to the formation of a robust abscess.

**Fig 5:**
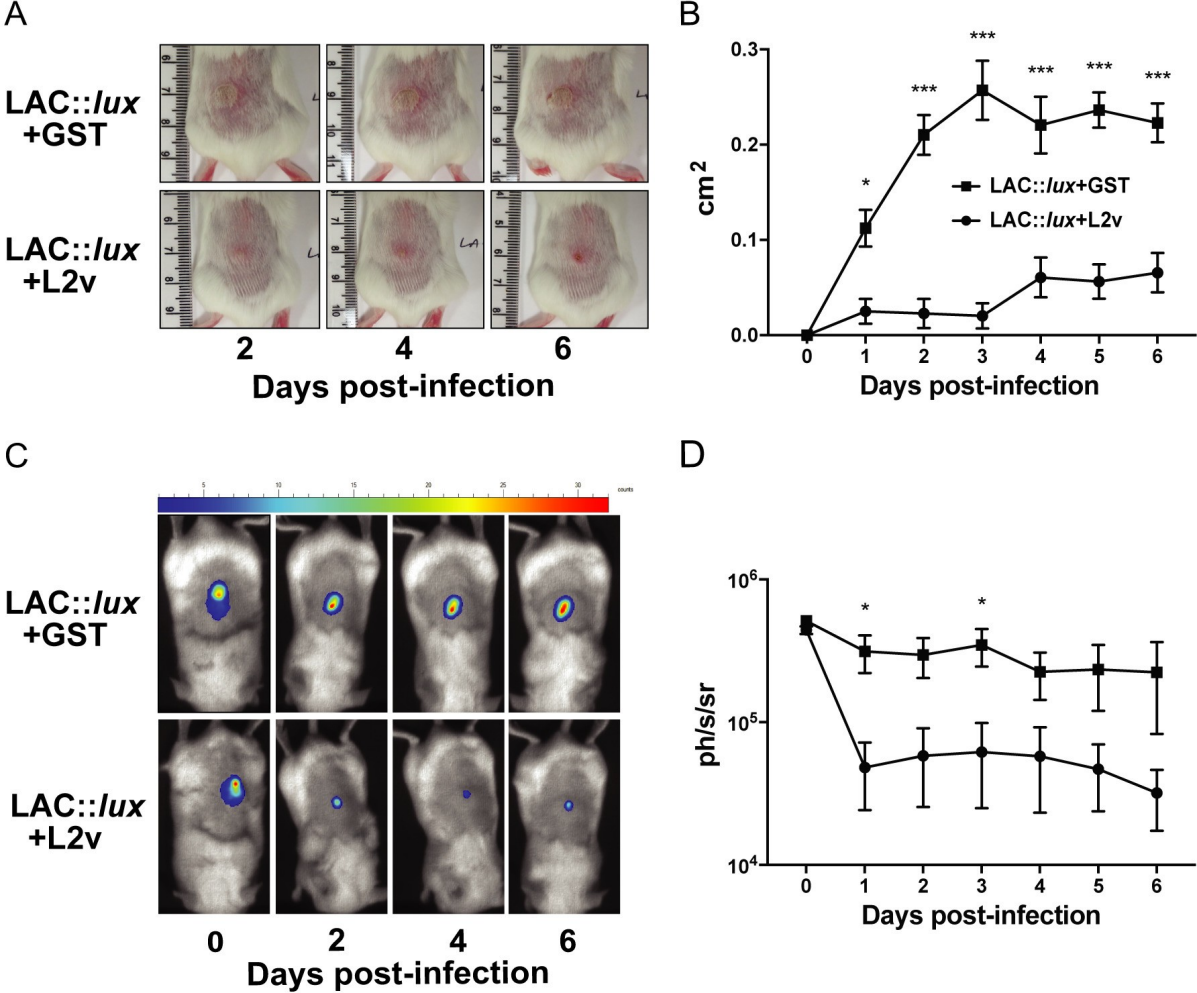
*In vivo* inhibition of ClfB-loricrin interaction leads to reduced abscess area and bacterial burden. BALB/c mice were infected subcutaneously with 2x10^7^ CFU *S. aureus* LAC::*lux* pre-incubated with loricrin loop 2 region (L2v) or GST (24 μM) and abscess lesion size and bacterial burden was measured. Representative lesions from the dorsal area of mice from each group are shown (A) and results are expressed as total lesion size (cm^2^) ± SEM (B). Bioluminescence imaging was carried out using a Photon Imager and representative *in vivo* bioluminescence images are shown (C). Results are expressed as mean total photon flux (photons per second per steradian) ± SEM (D). n = 8 per group. Data pooled from 2 independent experiments. Two-way ANOVA with Bonferroni post-test used to analyze differences between groups. * P < 0.05, *** P < 0.001.

Next, recombinant loricrin loop L2v-GST (L2v) was used to block the interaction between ClfB and its native ligands. LAC::*lux* (2x10^7^ CFU) was pre-incubated with L2v or GST prior to s.c. injection. Abscess lesion area was measured over the 6 day infection period and mice that received LAC::*lux*+L2v had reduced abscess lesion area compared to the LAC::*lux*+GST group (Fig 5A and 5B). In addition, bioluminescence signal was reduced throughout the infection period in LAC::*lux*+L2v infected mice compared to mice that received LAC::*lux*+GST (Fig 5C and 5D) and this reduction in bioluminescence was confirmed with a significant decrease in the bacterial burden in the skin on day 3 and 6 post-infection (S4 Fig).

### A model vaccine containing ClfB protects against *S*. *aureus* SSTI

**Fig 6:**
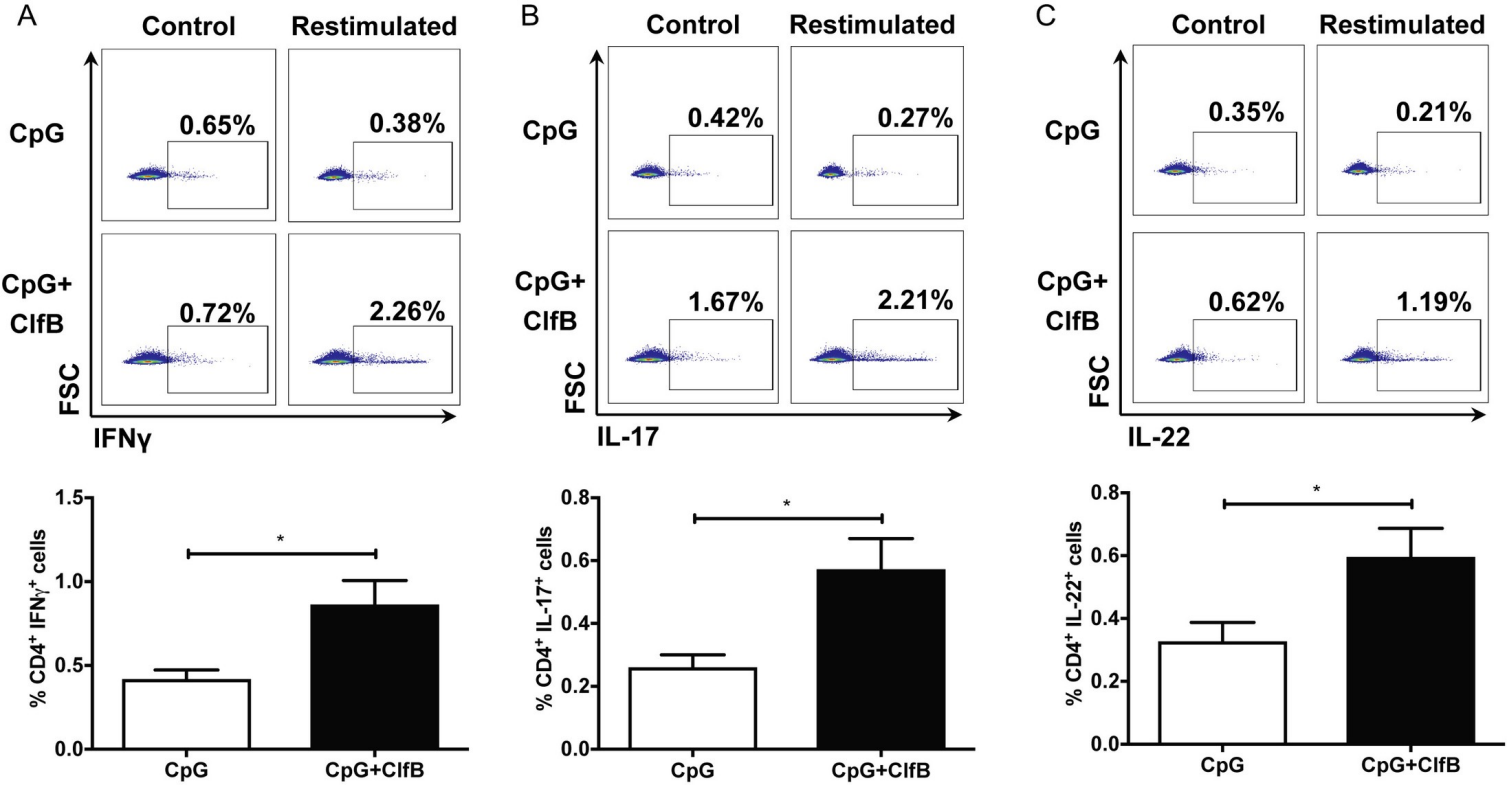
Vaccination with ClfB in combination with CpG leads to cellular immune responses. BALB/c mice were vaccinated subcutaneously with CpG (50μg/mouse) alone or in combination with ClfB (5μg/mouse) on day 0, 14, 28. Antigen-specific cellular immune responses were measured on day 42 by *ex vivo* stimulation of inguinal lymph node cells with ClfB (10μg/ml). The percentage of CD4^+^IFN𝛾^+^ (A), CD4^+^IL-17^+^ (B) and CD4^+^IL-22^+^ (C) cells within the CD45^+^CD3^+^ population was assessed by flow cytometry. Results expressed as mean percentage ± SEM. n = 5–6 per group. Mann-Whitney U test used to analyze differences between groups. * P < 0.05.

**Fig 7:**
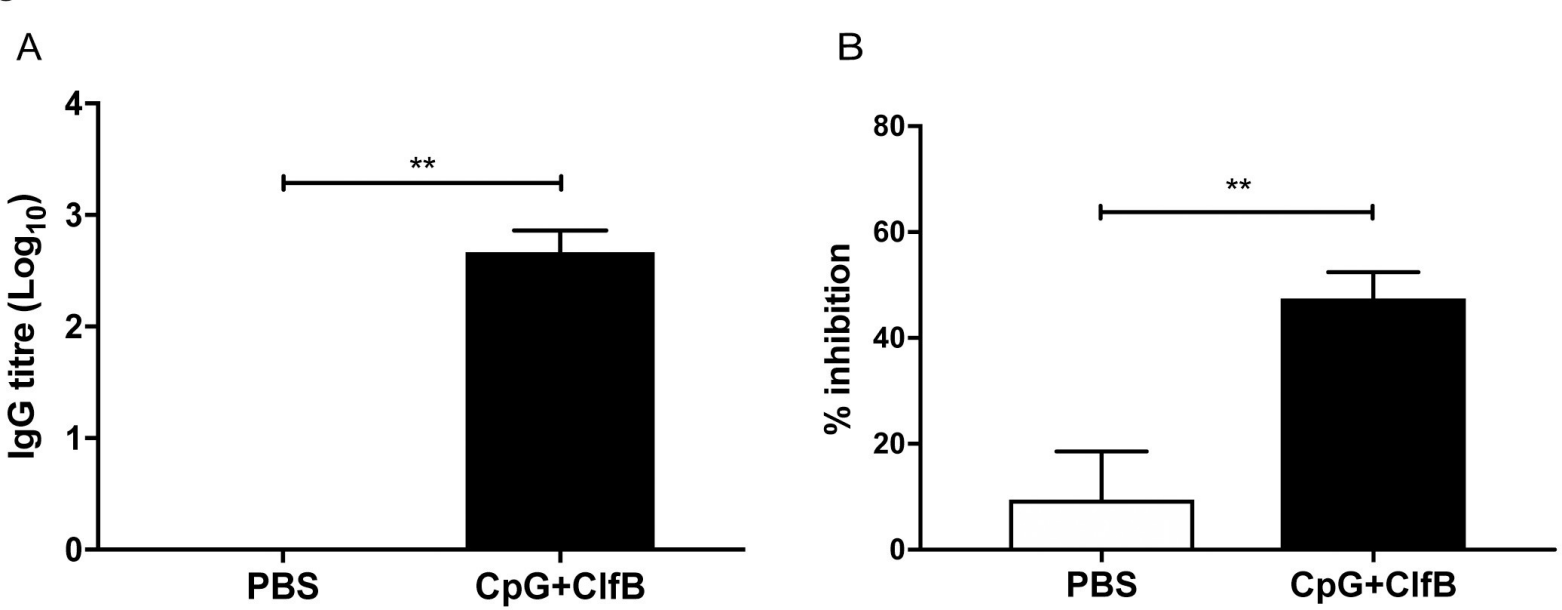
Vaccination with ClfB in combination with CpG induces functional blocking ClfB-specific antibodies. BALB/c mice were vaccinated subcutaneously with PBS or CpG in combination with ClfB (5μg/mouse) on day 0, 14, 28. Sera were collected on day 42 to assess antigen-specific humoral immune responses. ClfB-specific antibody titers were determined by ELISA and results are expressed as Log10 IgG titer (A). The presence of neutralizing antibodies was determined by measuring the ability of sera to inhibit the binding of *L. lactis* expressing ClfB (pKS80::clfB) to immobilised loricrin (B). n = 5 per group. Mann-Whitney U test used to analyze differences between groups. ** P < 0.01.

Having discovered a novel role for ClfB during *S*. *aureus* skin infection, the use of ClfB as a vaccine antigen targeted against SSTIs was investigated. A model vaccine containing ClfB was formulated with the Toll-like receptor 9 (TLR9) agonist CpG as an adjuvant. Naïve BALB/c mice were vaccinated s.c. with PBS, CpG alone or in combination with ClfB on day 0, 14, and 28 and challenged via s.c injection with LAC::*lux* on day 42. Antigen-specific cellular and humoral immune responses were assessed prior to challenge. Immunization with ClfB in combination with CpG drove a significant increase in ClfB-specific IFNγ (Fig 6A), IL-17 (Fig 6B) and IL-22 (Fig 6C) producing CD4^+^ T cells by inguinal lymph node (ILN) cells (skin-draining lymph nodes) compared to CpG-immunized mice. ClfB-specific CD8^+^ T cells producing IFNγ, IL-17 and IL-22 were also increased in the ILN of ClfB-vaccinated groups compared to the adjuvant alone group (S5 Fig). Vaccination with ClfB also induced significantly elevated ClfB-specific serum IgG titres in mice compared to the control group (Fig 7A). We confirmed that these antibodies were functional by investigating their ability to inhibit *L*. *lactis* expressing ClfB (pKS80:clfB [13]) to bind to immobilised loricrin (Fig 7B).

**Fig 8:**
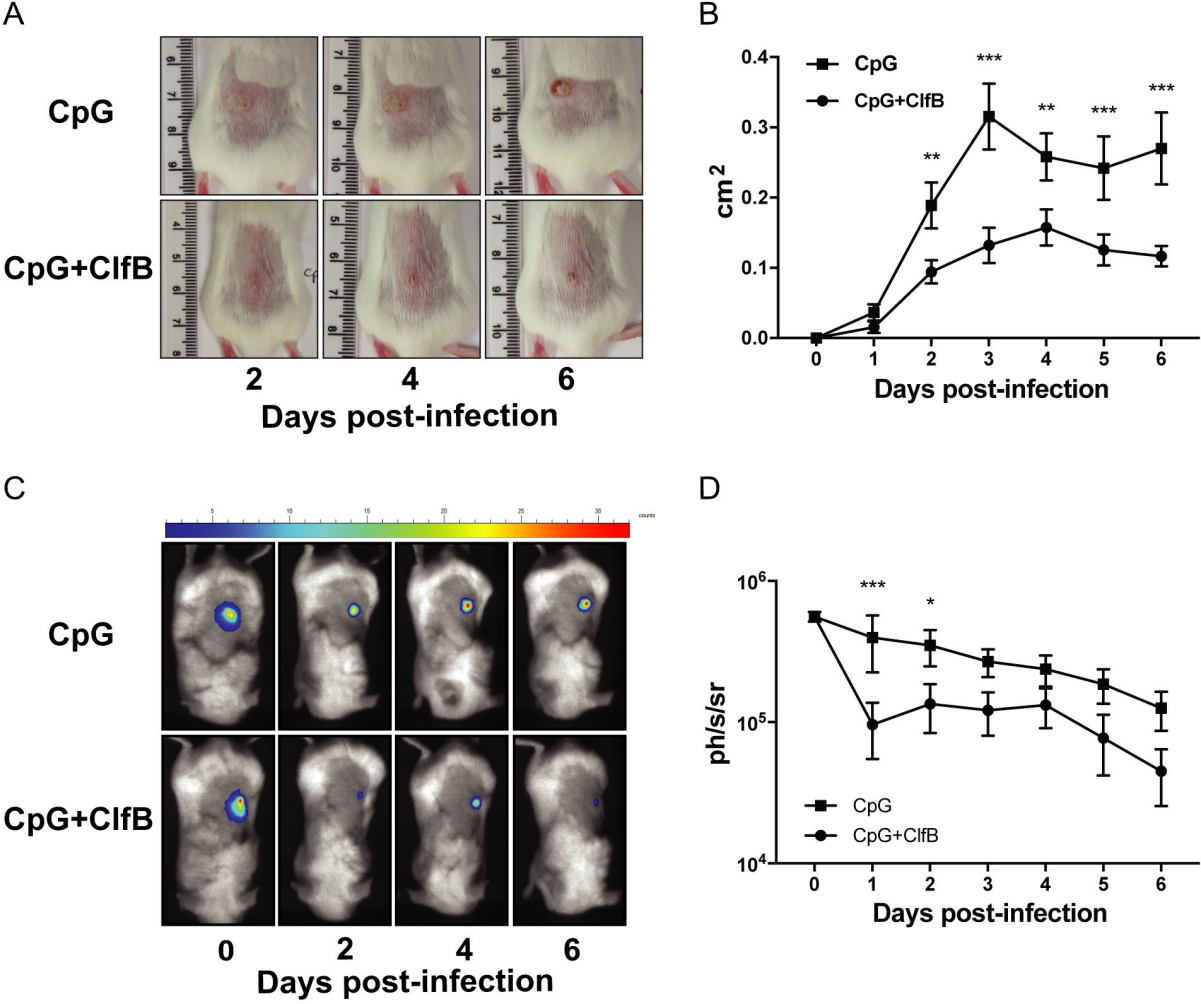
Vaccination with ClfB in combination with CpG offers protection against *S. aureus* SSTI. BALB/c mice were vaccinated subcutaneously with CpG (50μg/mouse) alone or in combination with ClfB (5μg/mouse) on day 0, 14, 28. On day 42, mice were infected subcutaneously with 2x10^7^ CFU *S. aureus* LAC::*lux* and abscess lesion size and bacterial burden was measured. Representative lesions from the dorsal area of mice from each group are shown (A) and results are expressed as total lesion size (cm^2^) ± SEM (B). Bioluminescence imaging was carried out using a Photon Imager and representative *in vivo* bioluminescence images are shown (C). Results are expressed as mean total photon flux (photons per second per steradian) ± SEM (D). Results are expressed as Log10 CFU/mg. n = 10 per group. Data pooled from 2 independent experiments. Two-way ANOVA with Bonferroni post-test used to analyze differences between groups. * P < 0.05, ** P < 0.01, *** P < 0.001.

Having demonstrated that immunisation with ClfB in combination with CpG could induce antigen-specific cellular and humoral immune responses, the ability of this vaccine to offer protection against *S*. *aureus* SSTIs was assessed. Mice vaccinated with ClfB in combination with CpG developed significantly smaller abscess lesions throughout the 6-day infection period compared to those vaccinated with adjuvant alone (Fig 8A and 8B) and the bacterial burden in the skin of ClfB vaccinated mice was decreased throughout the course of infection as measured by bioluminescence (Fig 8C and 8D). The reduction in bioluminescence signal was validated by quantifying the bacterial burden in the skin on day 6 post-infection, with a significant decrease in ClfB vaccinated mice compared to the adjuvant alone control (S6 Fig). Immunization with CpG alone offered no protection as compared to a PBS control group (S6 Fig).

Taken together, these results identify ClfB as an important vaccine antigen that can drive effective humoral and cellular immune responses, ultimately leading to protection against *S*. *aureus* SSTI.

## Discussion

Previous to this study, the main function attributed to ClfB was in facilitating *S*. *aureus* nasal colonization due to its ability to bind host ligands loricrin and cytokeratin 10 in the anterior nares [13–15]. This study identifies, for the first time, an important role for ClfB in the pathogenesis of SSTIs and provides proof-of-concept that ClfB represents a good candidate antigen for inclusion in next-generation vaccines targeting *S*. *aureus* SSTI. Using a *clfB* mutant in the clinically relevant CA-MRSA USA300 strain, LAC::*lux*, we demonstrate that ClfB is a virulence determinant during *S*. *aureus* skin infection. Crucially, ClfB appears to exert its effect early during the infection process during initial formation of the abscess.

Immunohistochemistry analysis revealed that loricrin, an important ligand for ClfB, was present in the outermost layer of the skin, the corneal layer, as expected; however, loricrin also appeared to be present within the wall of the skin abscess structure of LAC::*lux* infected mice. Although loricrin is normally confined to the granular and corneal layers of the skin [22, 23], the expression and localisation of loricrin is disrupted during wound healing [24, 25]. This may account for the presence of loricrin in the subcutaneous tissue. We propose that expression of loricrin within the damaged tissue facilitates the binding of *S*. *aureus* via ClfB which in turn promotes the development of the abscess. To investigate this proposed ClfB-loricrin interaction in the skin, loricrin deficient mice were used. Lor^-/-^ mice infected s.c. with LAC::*lux* formed significantly smaller abscess, with lower bacterial burden and reduced pathology compared to WT infected mice, suggesting that the interaction between *S*. *aureus* and loricrin is crucial during SSTIs. This effect appears to be ClfB-specific, and not due to ClfB binding to an alternative ligand in the skin, such as fibrinogen or keratin, as Lor^-/-^ mice infected with LAC::*lux* Δ*clfB* did not display any further significant reduction in abscess formation or the overall severity of skin infection. Furthermore, the interaction between *S*. *aureus* and loricrin could be blocked *in vivo* using recombinant loricrin. Pre-incubation with recombinant loricrin L2v abolished the ability of ClfB to bind to native ligands in the skin. Throughout the infection period, mice infected with LAC::*lux* pre-incubated with L2v had reduced bacterial burden and abscess lesion size compared to the control group.

These data reveal a novel, previously unidentified role for ClfB-loricrin binding in the pathogenesis of *S*. *aureus* SSTI; thus, targeting this pathway may represent a novel approach for the prevention of *S*. *aureus* SSTI. Interestingly, ClfB has also been shown to be a major player in mediating *S*. *aureus* adhesion to skin corneocytes in atopic dermatitis [26]. We therefore decided to investigate if vaccination with ClfB could protect against subsequent skin infection. Vaccination with ClfB has previously been shown to reduce murine nasal colonization [14], and vaccination with ClfB in combination with Freund’s adjuvant reduced bacterial burden in the kidneys of intravenously infected mice [27]. However, vaccination with ClfB has never previously been shown to protect against skin infection. In fact, only a handful of studies have attempted to vaccinate against *S*. *aureus* skin infection. These have included vaccination with ClfA [12], Als3p [28] and adenosine synthase A [29] in combination with alum and SasX combined with Freund’s adjuvant [30]. These vaccines have had variable efficacy, with only modest protection against SSTIs when ClfA [12] was used as vaccine antigen, supporting the notion that multiple CWA proteins may need to be targeted simultaneously, whereas Als3p [28] and adenosine synthase [29] offered significant protection. In the case of SasX [30], Freund’s adjuvant was used, limiting the potential for translation to humans. This study utilised the adjuvant, CpG, which due to its low reactogenicity and strong adjuvanticity is currently considered a prime adjuvant for future vaccines against infectious diseases [31]. In this current study, a model vaccine was formulated with ClfB in combination with CpG which has successfully driven Th1 and Th17 responses during immunization in mice [32–36].

Immunization with ClfB+CpG induced significant expansion of antigen-specific CD4^+^ and CD8^+^ T cells capable of producing IFNγ, IL-17 and IL-22. This correlates with our previous findings which demonstrates ClfB is capable of inducing antigen-specific T cell responses in human CD4^+^ T cells by inducing the activation of Th1 and Th17 cells [34]. IL-17 and IL-22 cytokine responses are known to be particularly important for protection during cutaneous infection with *S*. *aureus* through these cytokines ability to promote neutrophil recruitment to the infection site and also to drive local production of antimicrobial peptides, both important effector mechanisms for effective clearance of the bacterium [28, 37–40]. Vaccination with ClfB+CpG also activated humoral immune responses by inducing significant levels of ClfB-specific neutralizing antibodies compared to controls. This demonstrates that ClfB can activate both the humoral and cellular arms of the immune system which combined led to a substantial reduction in the severity of the subsequent LAC::*lux* skin infection, with reduced bacterial burden throughout the course of infection and a significant reduction in the abscess lesion area when compared to the adjuvant alone control group. Since ClfB is exposed on the bacterial cell surface and interacts directly with loricrin, we propose that antibodies produced in response to vaccination with ClfB may provide a protective effect by promoting opsonophagocytosis and by interfering with binding of ClfB to its ligand loricrin, which we have shown is present within the damaged skin and thus delays and prevents formation of a robust abscess structure. Importantly we demonstrated that direct blockade of the ClfB-loricrin interaction *in vivo* using recombinant L2v significantly inhibited pathogenesis in this model. Simultaneously the expansion of ClfB-specific T cells producing effector cytokines such as IFNγ, IL-17 and IL-22 and their downstream effects on phagocytes [41–43] and the induction of antimicrobial peptide expression [37, 44, 45] will directly contribute to the clearance of invading bacteria. Furthermore IL-22 has previously been shown to down regulate the expression of loricrin [46], which would reduce the levels available for ClfB to bind to thus further inhibiting abscess formation.

Taken together, the results of this study highlight the importance of ClfB as a virulence determinant during *S*. *aureus* SSTIs which plays a role in determining the bacterial burden at the site of infection but most importantly identifies for the first time how ClfB also affects the structure and formation of the skin abscess. ClfB exerts its effect in the early stage of infection and its interaction with loricrin appears to play a role during pathogenesis. This interaction is likely specific to the skin as previous studies have demonstrated no difference in systemic *S*. *aureus* infection in WT and Lor^-/-^ mice [13]. The data presented herein therefore support the targeting of ClfB in future vaccines specifically for the prevention of *S*. *aureus* SSTIs. These vaccines will likely be multivalent and will potentially include multiple cell wall-anchored proteins in addition to secreted proteins, and will be required to drive protective cellular in addition to humoral responses.

## Materials and methods

### Bacterial growth conditions and strain construction

*S*. *aureus* was grown on tryptic soy agar (TSA; Oxoid) or in tryptic soy broth (TSB; Oxoid) at 37°C. *S*. *aureus* strain USA300 LAC::*lux* has been previously described [47]. LAC::*lux* was transformed with plasmid DNA isolated from *Escherichia coli* strain IM08B [48] by electroporation [49]. Deletion of the *clfB* gene was achieved by allelic exchange using the plasmid pIMAY [50] as previously described [13]. The *clfB* mutation in LAC::*lux* Δ*clfB* was confirmed by DNA sequencing of a PCR amplimer. LAC::*lux* Δ*clfB* was phenotypically indistinguishable from LAC::*lux* in terms of growth rate and haemolysis on sheep blood agar. LAC::*lux* Δ*clfB* was unable to bind to recombinant loricrin. Complementation of the mutant was achieved by transformation with the plasmid pCU1::*clfB* [51]. This restored binding to loricrin to levels similar to that seen with the wild-type strain LAC::lux, while transformation with empty plasmid pCU1 [52] did not. (S7 Fig).

### Mice

Female BALB/c mice were obtained from Charles River Laboratories UK. Female wild-type FVB (WT) mice were obtained from Envigo UK. FVB Loricrin knockout (Lor^-/-^) mice have been previously described [53] and were bred in-house at Trinity College Dublin. All mice were used at 6–8 weeks. Mice were housed under specific pathogen-free conditions at the Trinity College Dublin Comparative Medicines unit. All animal experiments were conducted in accordance with the recommendations and guidelines of the health product regulatory authority (HPRA), the competent authority in Ireland and in accordance with protocols approved by Trinity College Dublin Animal Research Ethics Committee.

### Protein purification

Recombinant ClfB (amino acids 44 to 542) [16] was purified from *E*. *coli* by Ni^2+^ affinity chromatography as previously described [54]. Endotoxin was removed from the protein using Detoxi-Gel endotoxin-removing columns (Thermo Scientific). Recombinant GST-tagged L2v (GST-L2v) was purified from *E*. *coli* as previously described [13] using a GSTrap FF purification column (GE Healthcare), according to the manufacturer's instructions.

### Murine subcutaneous abscess model

The dorsal area of mice were shaved and injected s.c. with *S*. *aureus* (2x10^7^ CFU) in 100 μl of sterile PBS. For *in vivo* bioluminescence imaging, mice were anaesthetized via inhalation of 2% isoflurane (Iso-Vet) and imaged using the Biospace Lab PhotonIMAGER system. Bioluminescent activity was computed using M3 Vision software (Biospace Lab) and is presented as photons per second per steradian which represents actively metabolizing bacteria. Measurements of abscess lesion area were made by analysing digital photographs using M3 Vision software and pictures contain a millimetre ruler as a reference. To confirm that *in vivo* bioluminescent signals reflected bacterial burden, 8mm punch biopsies of lesional skin were taken at day 3 and 6 post-infection. Tissue was homogenized in sterile PBS and total bacterial burden was determined by plating out serial dilutions on TSA.

For *in vivo* blocking studies, LAC::*lux* (2x10^7^ CFU) was pre-incubated with recombinant GST or recombinant loricrin region 2v (L2v-GST; 24 μM)[13] for 30 min at RT before administration s.c. *in vivo* bioluminescence imaging was used to monitor the infection as previously described. Prior to *in vivo* challenge we validated that incubation of LAC::*lux* with L2v for 24 hours had no effect on viability.

### Murine immunization model

Naïve BALB/c mice were vaccinated via s.c. injection with PBS, CpG (50 μg/mouse, Hycult Biotech) alone, or in combination with ClfB (5 μg/mouse) on day 0, 14, and 28. Prior to challenge, on day 42, ILNs were collected for antigen recall and blood samples were collected for analysis of antibody titres. On day 42, mice were challenged with LAC::*lux* via s.c. injection (2x10^7^ CFU) and bioluminescent imaging and abscess lesion area measurements were carried out.

### Measuring the cellular and humoral responses to ClfB

Lymphocytes were isolated from the ILN on day 42 post-immunization and restimulated *in vitro* with ClfB (10 μg/ml) in cRPMI for 6 h. cRPMI comprised RPMI (Sigma-Aldrich), 10% (vol/vol) fetal calf serum (Biosera), 100 mM L-glutamine (Gibco) and 100 μg/ml penicillin-streptomycin (Gibco). Brefeldin A (5 μg/ml) was added to the cultures for 5 h before surface staining with fluorochrome-conjugated antibodies against CD45 (eBioscience, clone 30-F11), CD3 (Biolegend, clone 17A2), CD4 (eBioscience, clone GK1.5) and CD8 (eBioscience, clone 53–6.7). The cells were fixed and permeabilised using Dako IntraStrain Fixation and Permeabilisation kit, followed by intracellular staining with against IFNγ (eBioscience, clone XMG1.2), IL-17A (eBioscience, eBio17B7) and IL-22 (eBioscience, clone IH8PWSR). Flow cytometric data were acquired with a BD FACSCanto II and analysed using FlowJo software (Tree Star Inc.).

ClfB-specific IgG antibody titres in sera were quantified by sandwich ELISA using anti-murine IgG (1 in 4000; Sigma-Aldrich), as previously described [55]. Antibody concentrations were expressed as endpoint titres calculated by regression curve of OD values versus reciprocal serum levels to a cut-off point of 2 standard deviations above control serum. The ability of mouse serum to neutralise the ligand binding activity of ClfB was tested by incubating *L*. *lactis* pKS80::clfB with serum for 30 min prior to adding the bacteria to wells coated with GST-L2v and incubating at 37 C for 1.5 h. Wells were washed with PBS and adherent cells fixed with formaldehyde (25% v/v), stained with crystal violet and the A570 measured. Adherence was calculated as a percentage of adherence in the absence of serum and percentage inhibition was determined by subtracting adherence percentage from 100.

### Histopathology analysis

Skin abscess tissue was excised and fixed in 10% formalin and embedded in paraffin. Sections (5–10 μm) were mounted onto glass slides and stained with haematoxylin and eosin. Tissue samples were imaged by bright field microscopy using an Olympus BX51 microscope. To quantify differences between groups, a histology scoring system was devised which differentiated abscesses according to their architecture. Sections were scored by three independent blinded observers. Abscess area was measured and scored according to size (S2 Table).

For loricrin staining, sections were incubated with blocking buffer (1% BSA, 10% goat serum) for 1 h. Sections were incubated overnight at 4°C with primary antibody (anti-mouse loricrin, Covance). Endo-peroxidase activity was blocked by incubation in 3% hydrogen peroxide solution, followed by incubation for 30 min with secondary antibody conjugated with horseradish peroxidase (HRP). Controls were stained with secondary antibody alone (S2 Fig). Colorimetric development was achieved using 3,3’-diaminobenzidine (Vector Labs) and counterstained with haematoxylin.

### Statistical analysis

Statistical analyses were performed using GraphPad Prism software. Differences between groups were analyses using either Mann-Whitney U test or Two-way ANOVA with Bonferroni post-test. A *p* value <0.05 was considered significant.

### Ethics statement

All animal experiments were conducted in accordance with the recommendations and guidelines of the health product regulatory authority (HPRA), the competent authority in Ireland and in accordance with protocols approved by Trinity College Dublin Animal Research Ethics Committee. Project authorisation number AE19136/P006. Euthanasia by CO_2_ inhalation.

## Supporting information

S1 Fig The absence of ClfB or loricrin does not affect initial bacterial burden in the skin at 6 hours post-infection. Wild-type FVB (WT) and Lor^-/-^ mice were infected subcutaneously with 2x10^7^ CFU *S*. *aureus* LAC::*lux* or LAC::*lux* Δ*clfB* and bacterial burden in the skin was assessed by viable counting at 6 hours post-infection. Results are expressed as Log_10_ CFU/mg. n = 3 per group.
(PDF)

S2 Fig Loricrin staining within the skin abscess tissue of LAC::*lux* Δcl*fB* infected mice. BALB/c mice were infected subcutaneously with 2x10^7^ CFU *S*. *aureus* LAC::*lux* Δ*clfB* and abscess tissue was excised at 48h post-infection. Tissue was fixed, embedded in paraffin wax and sectioned before anti-loricrin staining (A) or secondary antibody only control (B) was carried out. Black arrows indicate the presence of loricrin in the abscess wall structure (A). Representative images of n = 2 stained sections.
(TIF)

S3 Fig Loricrin-ClfB interaction is critical for abscess formation. Wild-type FVB (WT) and Lor^-/-^ mice were infected subcutaneously with 2x10^7^ CFU *S*. *aureus* LAC::*lux* or LAC::*lux* Δ*clfB*. and abscess tissue was excised at 96 hours. Tissue was fixed, embedded in paraffin wax and sectioned before haematoxylin and eosin staining was performed. Representative sections from each group are shown. n = 3 per group.
(TIF)

S4 Fig Blocking the ligand binding ability of ClfB reduced bacterial burden during *S. aureus* SSTI. BALB/c mice were infected subcutaneously with 2x10^7^ CFU *S*. *aureus* LAC::*lux* pre-incubated with loricrin loop 2 region (L2v) or GST bacterial burden was measured. Bacterial burden in the skin was assessed by viable counting on day 3 (A) and day 6 (B) post-infection. Results are expressed as Log_10_ CFU/mg. n = 8 per group. Data pooled from 2 independent experiments. Mann-Whitney U test used to analyze differences between groups. *** *P* < 0.001.
(TIF)

S5 Fig Vaccination with ClfB in combination with CpG leads to CD8^+^ cellular immune responses. BALB/c mice were vaccinated subcutaneously with CpG (50μg/mouse) alone or in combination with ClfB (5μg/mouse) on day 0, 14, 28. Antigen-specific cellular immune responses were measured on day 42 by *ex vivo* stimulation of inguinal lymph node cells with ClfB (10μg/ml). The percentage of CD8^+^IFN𝛾^+^ (A), CD8^+^IL-17^+^ (B) and CD8^+^IL-22^+^ (C) cells within the CD45^+^CD3^+^ population was assessed by flow cytometry. Results expressed as mean percentage ± SEM. n = 6 per group. Mann-Whitney U test used to analyze differences between groups. * *P* < 0.05.
(TIF)

S6 Fig Vaccination with ClfB in combination with CpG reduces the bacterial burden in the skin during *S. aureus* SSTI. BALB/c mice were vaccinated subcutaneously with PBS, CpG (50μg/mouse) alone or in combination with ClfB (5μg/mouse) on day 0, 14, 28. On day 42, mice were infected subcutaneously with 2x10^7^ CFU *S*. *aureus* LAC::*lux* and bacterial burden was measured. Bacterial burden in the skin was assessed by viable counting on day 6 post-infection. Results are expressed as Log_10_ CFU/mg. n = 10 per group. Data pooled from 2 independent experiments. Mann-Whitney U test used to analyze differences between groups. * *P* < 0.05.
(TIF)

S7 Fig LAC::*lux* Δcl*fB* is unable to adhere to immobilized loricrin. Microtiter plates were coated with GST-tagged loricrin loop 2 region (L2v, 0.3125 μg/ml). Adherence of *S*. *aur*eus grown to exponential phase to immobilized L2v was assessed by staining with crystal violet and measuring absorbance at 570nm. Data pooled from 3 independent experiments. Error bars represent the standard deviation. One-way ANOVA with Tukeys post-test used to analyze differences between groups. *** *P* < 0.001.
(TIF)

S1 Table Dissemination of LAC::*lux* to peripheral organs at 24h post-infection. Wild-type FVB (WT) and Lor^-/-^ mice were infected subcutaneously with 2x10^7^ CFU *S*. *aureus* LAC::*lux* or LAC::*lux* Δ*clfB* and bacterial burden in the blood and peripheral organs assessed by viable counting at 24 hours post-infection. Results are expressed as Log_10_ CFU/mg. n = 3 per group.
(TIF)

S2 Table Abscess area scoring system. (TIF)
