# Supplementary material for: Clumping factor B is an important virulence factor during Staphylococcus aureus skin infection and a promising vaccine target
Source: PLoS Pathog. 2019 Apr 22;15(4):e1007713. doi: 10.1371/journal.ppat.1007713 (PMC6497315; doi:10.1371/journal.ppat.1007713)
Supplement: S1 Fig — Wild-type FVB (WT) and Lor-/- mice were infected subcutaneously with 2x10^7^ CFU S. aureus LAC::lux or LAC::lux ΔclfB and bacterial burden in the skin was assessed by viable counting at 6 hours post-infection. Results are expressed as Log10 CFU/mg. n = 3 per group. (PDF) [file ppat.1007713.s001.pdf]

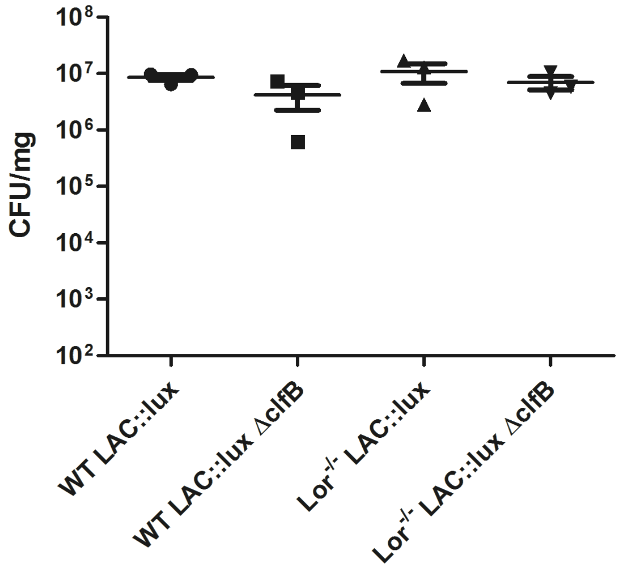

Supplemental Figure 1. The absence of ClfB or Loricrin does not affect initial bacterial burden in the skin at 6 hours post-infection. Wild-type FVB (WT) and $Lor^{-/-}$ mice were infected subcutaneously with $2x10^7$ CFU *S. aureus* LAC::*lux* or LAC::*lux* Δ*clfB* and bacterial burden in the skin was assessed by viable counting at 6 hours post-infection. Results are expressed as Log_{10} CFU/mg. n=3 per group.

	WT LAC::lux	WT LAC::lux ΔclfB	Lor$^{-/-}$ LAC::lux	Lor$^{-/-}$ LAC::lux ΔclfB
**Liver**	2.92±0.14	2.70±0.43	2.76±0.41	3.05±0.34
**Kidneys**	2.98±0.39	2.32±0.22	3.04±0.49	2.68±0.24
**Spleen**	2.09±1.08	1.70±0.85	0.47±0.47	0
**Blood**	0	0	0	0

Supplemental Table 1: Dissemination of LAC:*lux* to peripheral organs at 24h post-infection. Wild-type FVB (WT) and Lor$^{-/-}$ mice were infected subcutaneously with $2x10^7$ CFU *S. aureus* LAC::*lux* or LAC::*lux* Δ*clfB* and bacterial burden in the blood and peripheral organs assessed by viable counting at 24 hours post-infection. Results are expressed as Log_{10} CFU/mg. n=3 per group.

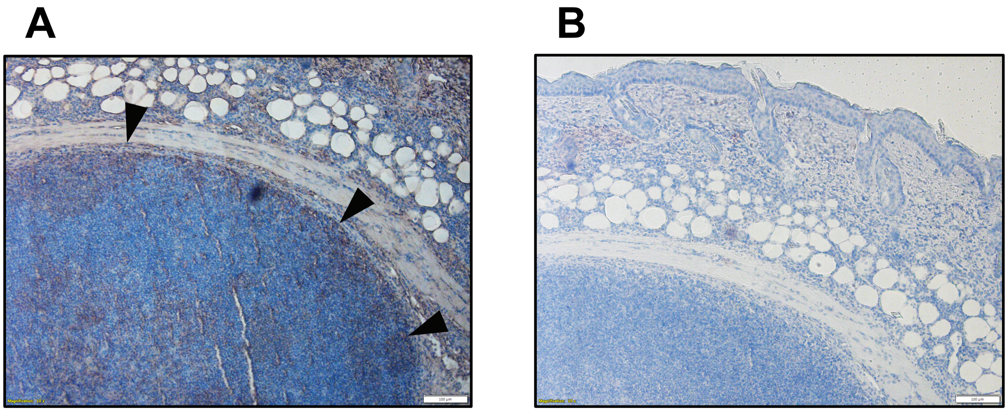

Supplemental Figure 2. Loricrin staining within the skin abscess tissue of LAC::*lux* Δ*clfB* infected mice. BALC/c mice were infected subcutaneously with $2x10^7$ CFU *S. aureus* LAC::*lux* Δ*clfB* and abscess tissue was excised at 48h post-infection. Tissue was fixed, embedded in paraffin wax and sectioned before anti-loricrin staining (A) or secondary antibody only control (B) was carried out. Black arrows indicate the presence of loricrin in the abscess wall structure (A). Representative images of n=2 stained sections.

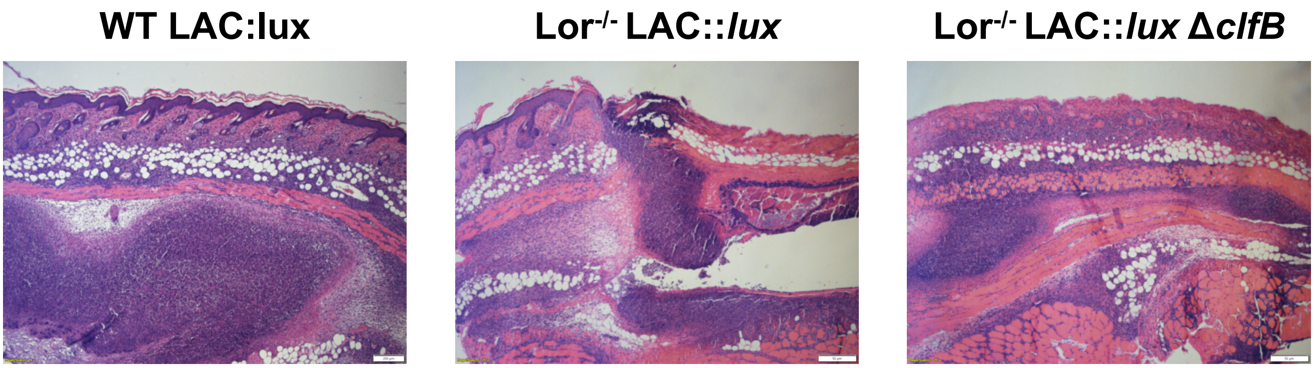

Supplemental Figure 3. Loricrin-ClfB interaction is critical for abscess formation. Wild-type FVB (WT) and $Lor^{-/-}$ mice were infected subcutaneously with $2x10^7$ CFU *S. aureus* LAC::*lux* or LAC::*lux* Δ*clfB.* and abscess tissue was excised at 96 hours. Tissue was fixed, embedded in paraffin wax and sectioned before haematoxylin and eosin staining was performed. Representative sections from each group are shown. N=3 per group

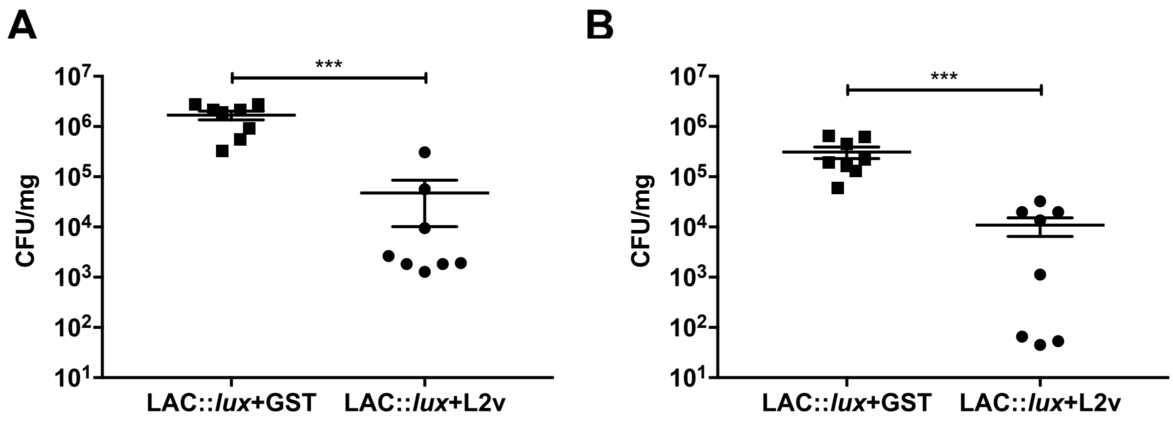

Supplemental Figure 4. Blocking the ligand binding ability of ClfB reduced bacterial burden during *S. aureus* SSTI. BALC/c mice were infected subcutaneously with $2x10^7$ CFU *S. aureus* LAC::*lux* pre-incubated with loricrin loop 2 region (L2v) or GST bacterial burden was measured. Bacterial burden in the skin was assessed by viable counting on day 3 (A) and day 6 (B) post-infection. Results are expressed as Log_{10} CFU/mg. n=8 per group. Data pooled from 2 independent experiments. Mann-Whitney U test used to analyze differences between groups. *** $P < 0.001$.

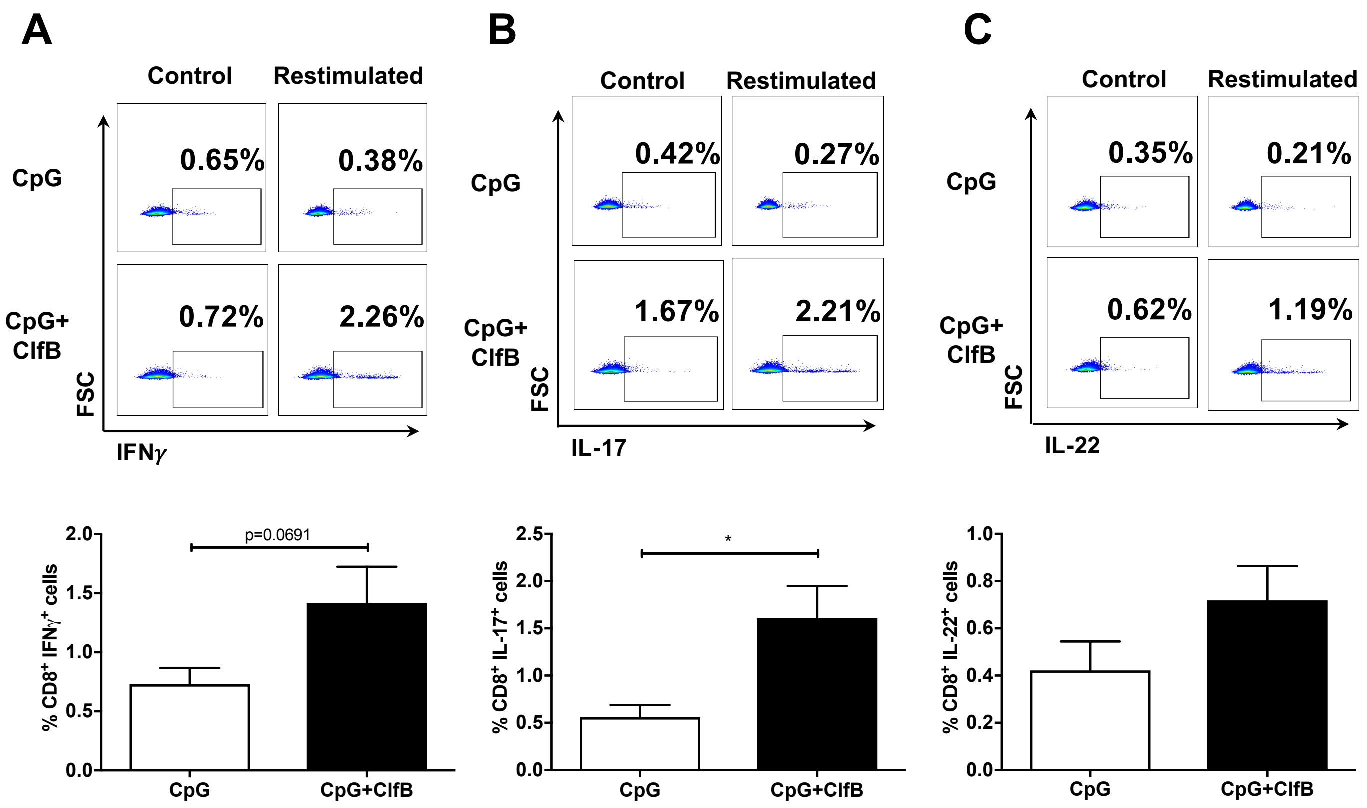

Supplemental Figure 5. Vaccination with ClfB in combination with CpG leads to CD8$^+$ cellular immune responses. BALC/c mice were vaccinated subcutaneously with CpG (50µg/mouse) alone or in combination with ClfB (5µg/mouse) on day 0, 14, 28. Antigen-specific cellular immune responses were measured on day 42 by *ex vivo* stimulation of inguinal lymph node cells with ClfB (10µg/ml). The percentage of CD8$^+$IFNγ^+ (A), CD8$^+$IL-17$^+$ (B) and CD8$^+$IL-22$^+$ (C) cells within the CD45$^+$CD3$^+$ population was assessed by flow cytometry. Results expressed as mean percentage ± SEM. Sera was collected on day 42 to assess antigen-specific humoral immune responses. ClfB-specific antibody titres were determined by ELISA and results are expressed as Log10 IgG titre (G). n=6 per group. Mann-Whitney U test used to analyze differences between groups. * $P < 0.05$.

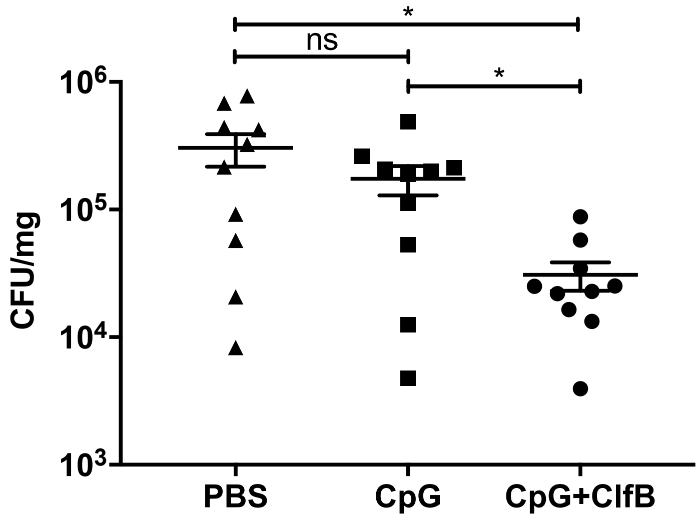

Supplemental Figure 6. Vaccination with ClfB in combination with CpG reduces the bacterial burden in the skin during *S. aureus* SSTI. BALC/c mice were vaccinated subcutaneously with PBS, CpG (50μg/mouse) alone or in combination with ClfB (5μg/mouse) on day 0, 14, 28. On day 42, mice were infected subcutaneously with 2×10^7 CFU *S. aureus* LAC::*lux* and bacterial burden was measured. Bacterial burden in the skin was assessed by viable counting on day 6 post-infection. Results are expressed as Log_{10} CFU/mg. n=10 per group. Data pooled from 2 independent experiments. Mann-Whitney U test used to analyze differences between groups. * $P < 0.05$.

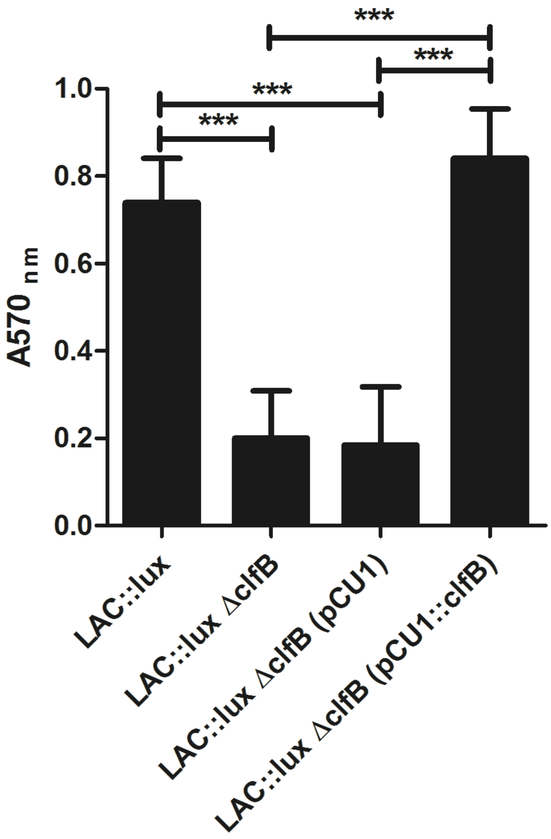

Supplemental Figure 7. LAC::*lux* Δ*clfB* is unable to adhere to immobilized loricrin. Microtiter plates were coated with GST-tagged loricrin loop 2 region (L2v, 0.3125 µg/ml). Adherence of *S. aur*eus grown to exponential phase to immobilized L2v was assessed by staining with crystal violet and measuring absorbance at 570nm. Data pooled from 3 independent experiments. Error bars represent the standard deviation. One-way ANOVA with Tukeys post-test used to analyze differences between groups. *** $P < 0.001$.

Score	Area
1	< 0.7 mm^2
2	0.7 mm^2 – 1.4 mm^2
3	1.4 mm^2 – 2.1 mm^2
4	2.1 mm^2 – 2.8 mm^2
5	> 2.8 mm^2

Supplemental Table 2. **Abscess area scoring system.**
