# Supplementary figures and images for: Clumping factor B is an important virulence factor during Staphylococcus aureus skin infection and a promising vaccine target
Source: PLoS Pathog. 2019 Apr 22;15(4):e1007713. doi: 10.1371/journal.ppat.1007713 (PMC6497315; doi:10.1371/journal.ppat.1007713)

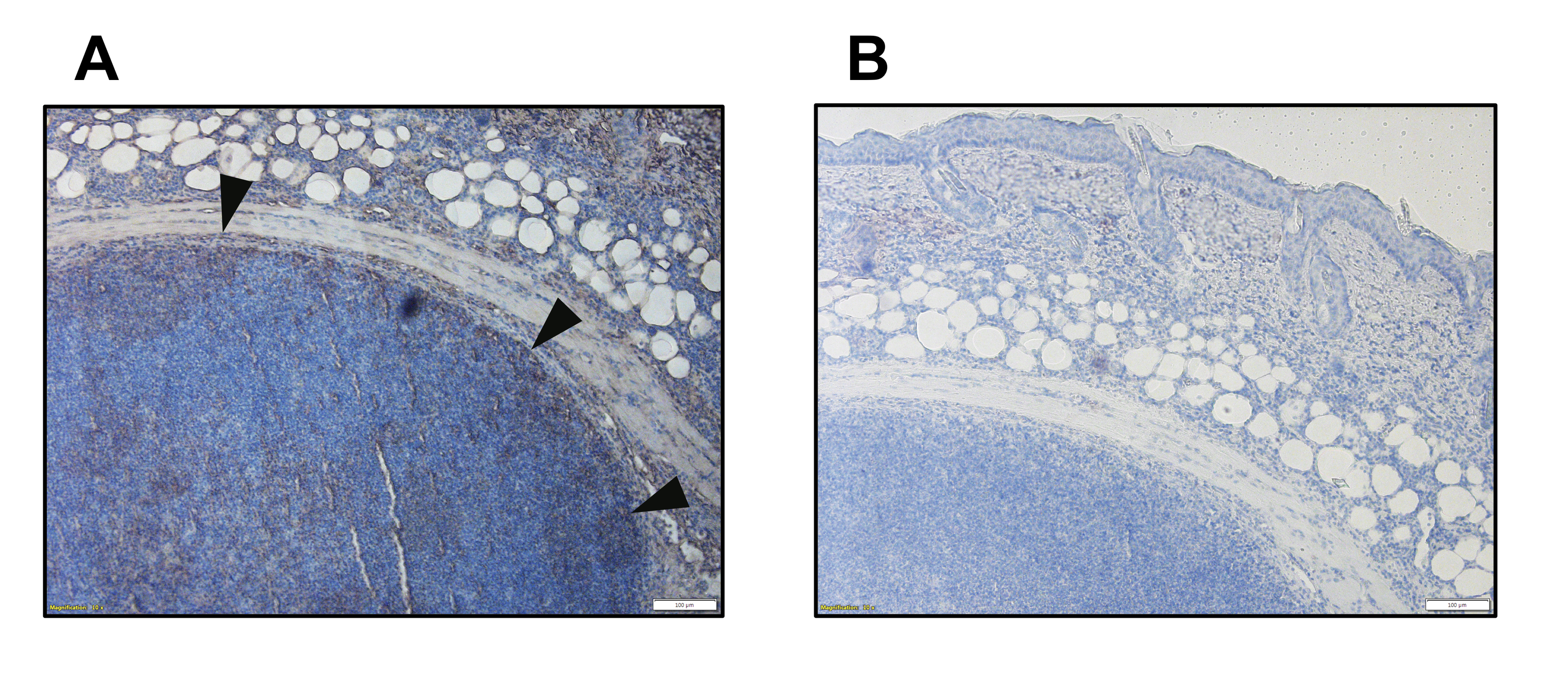

Supplement: S2 Fig — BALB/c mice were infected subcutaneously with 2x10^7^ CFU S. aureus LAC::lux ΔclfB and abscess tissue was excised at 48h post-infection. Tissue was fixed, embedded in paraffin wax and sectioned before anti-loricrin staining (A) or secondary antibody only control (B) was carried out. Black arrows indicate the presence of loricrin in the abscess wall structure (A). Representative images of n = 2 stained sections. (TIF) [file ppat.1007713.s002.tif]

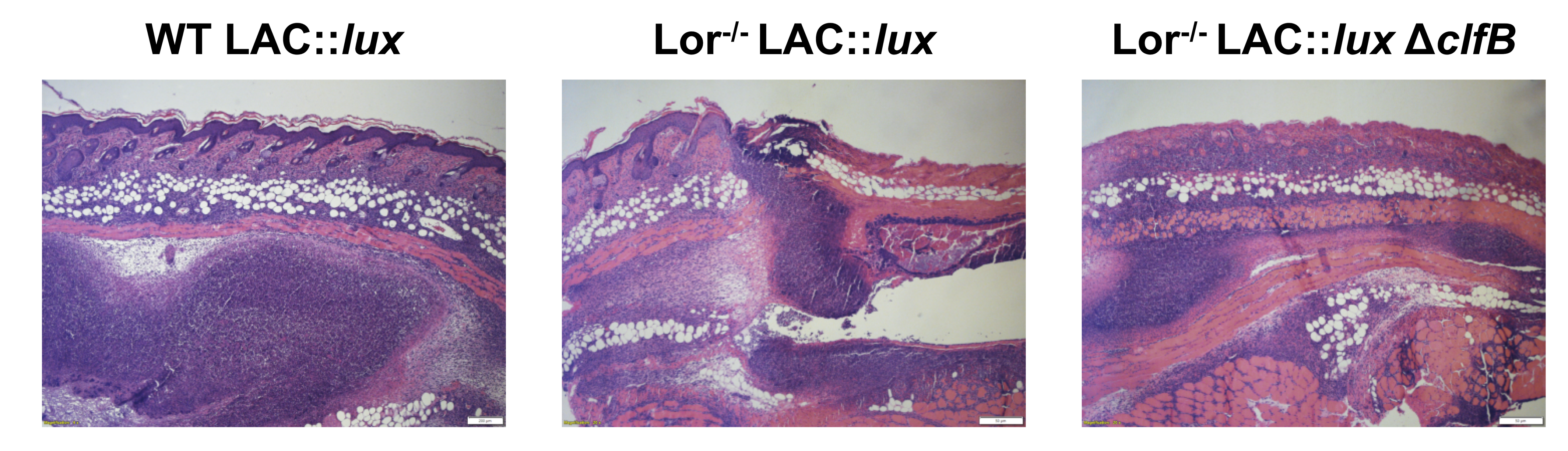

Supplement: S3 Fig — Wild-type FVB (WT) and Lor-/- mice were infected subcutaneously with 2x10^7^ CFU S. aureus LAC::lux or LAC::lux ΔclfB. and abscess tissue was excised at 96 hours. Tissue was fixed, embedded in paraffin wax and sectioned before haematoxylin and eosin staining was performed. Representative sections from each group are shown. n = 3 per group. (TIF) [file ppat.1007713.s003.tif]

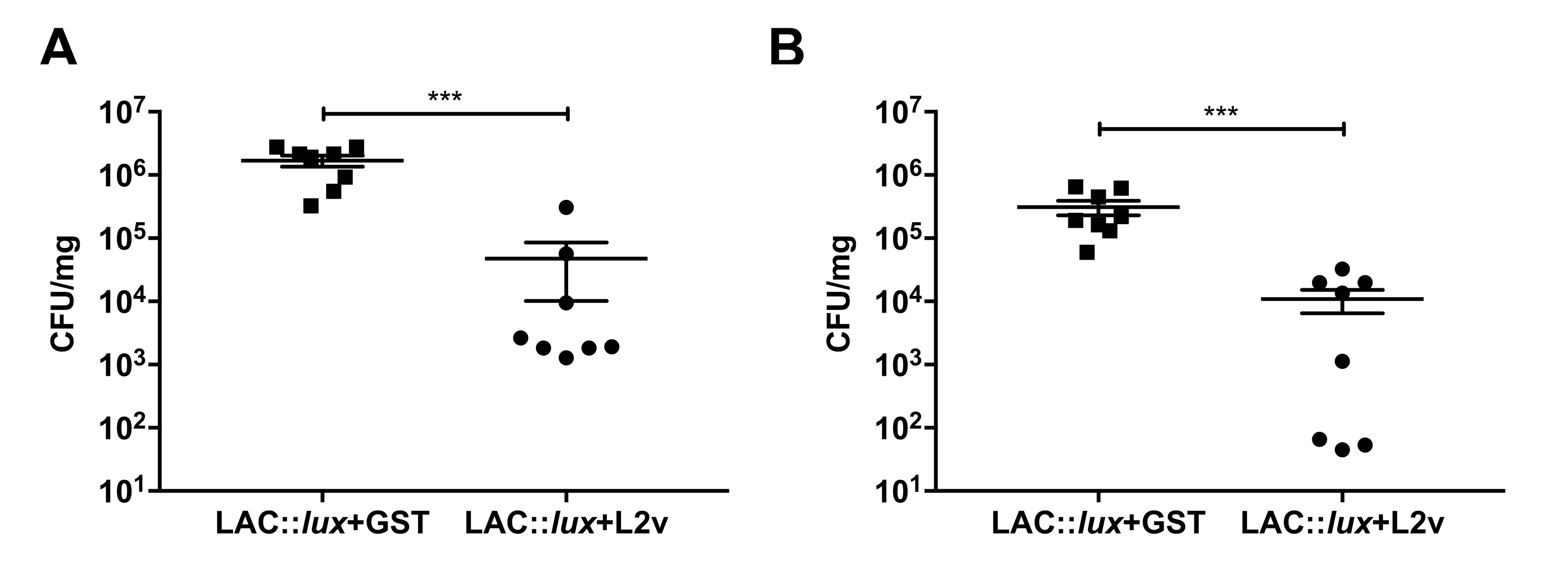

Supplement: S4 Fig — BALB/c mice were infected subcutaneously with 2x10^7^ CFU S. aureus LAC::lux pre-incubated with loricrin loop 2 region (L2v) or GST bacterial burden was measured. Bacterial burden in the skin was assessed by viable counting on day 3 (A) and day 6 (B) post-infection. Results are expressed as Log10 CFU/mg. n = 8 per group. Data pooled from 2 independent experiments. Mann-Whitney U test used to analyze differences between groups. *** P < 0.001. (TIF) [file ppat.1007713.s004.tif]

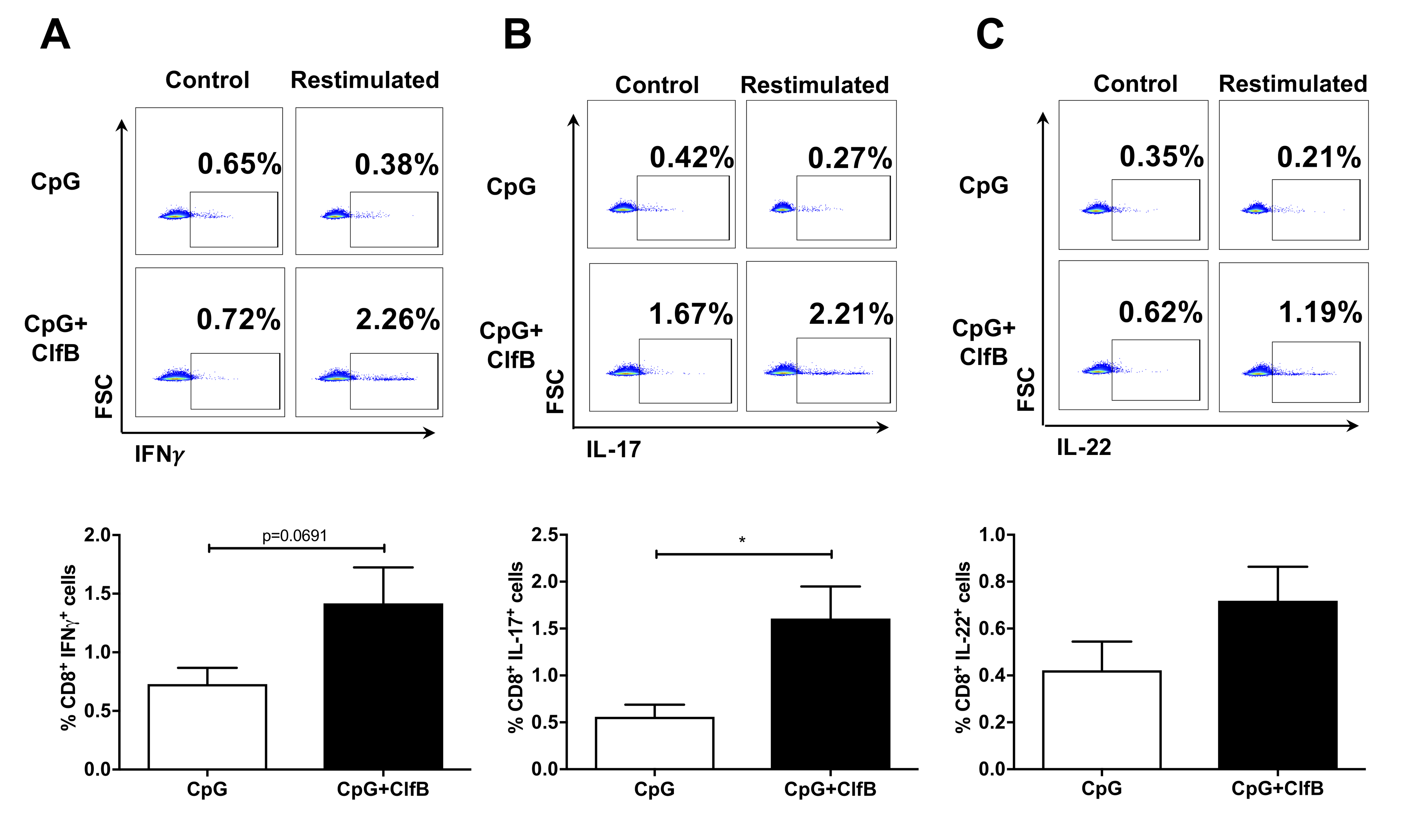

Supplement: S5 Fig — BALB/c mice were vaccinated subcutaneously with CpG (50μg/mouse) alone or in combination with ClfB (5μg/mouse) on day 0, 14, 28. Antigen-specific cellular immune responses were measured on day 42 by ex vivo stimulation of inguinal lymph node cells with ClfB (10μg/ml). The percentage of CD8+IFN𝛾+ (A), CD8+IL-17+ (B) and CD8+IL-22+ (C) cells within the CD45+CD3+ population was assessed by flow cytometry. Results expressed as mean percentage ± SEM. n = 6 per group. Mann-Whitney U test used to analyze differences between groups. * P < 0.05. (TIF) [file ppat.1007713.s005.tif]

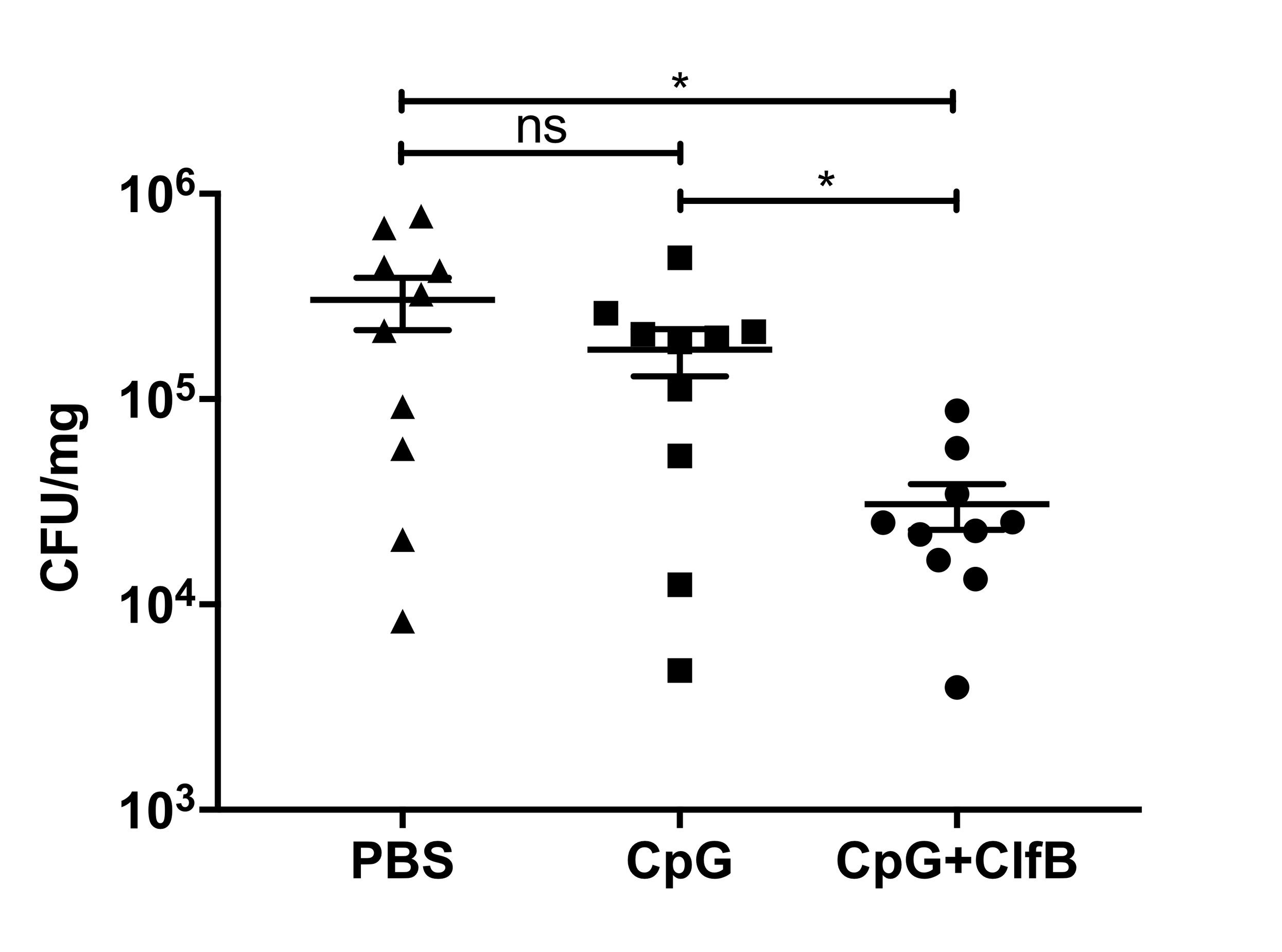

Supplement: S6 Fig — BALB/c mice were vaccinated subcutaneously with PBS, CpG (50μg/mouse) alone or in combination with ClfB (5μg/mouse) on day 0, 14, 28. On day 42, mice were infected subcutaneously with 2x10^7^ CFU S. aureus LAC::lux and bacterial burden was measured. Bacterial burden in the skin was assessed by viable counting on day 6 post-infection. Results are expressed as Log10 CFU/mg. n = 10 per group. Data pooled from 2 independent experiments. Mann-Whitney U test used to analyze differences between groups. * P < 0.05. (TIF) [file ppat.1007713.s006.tif]

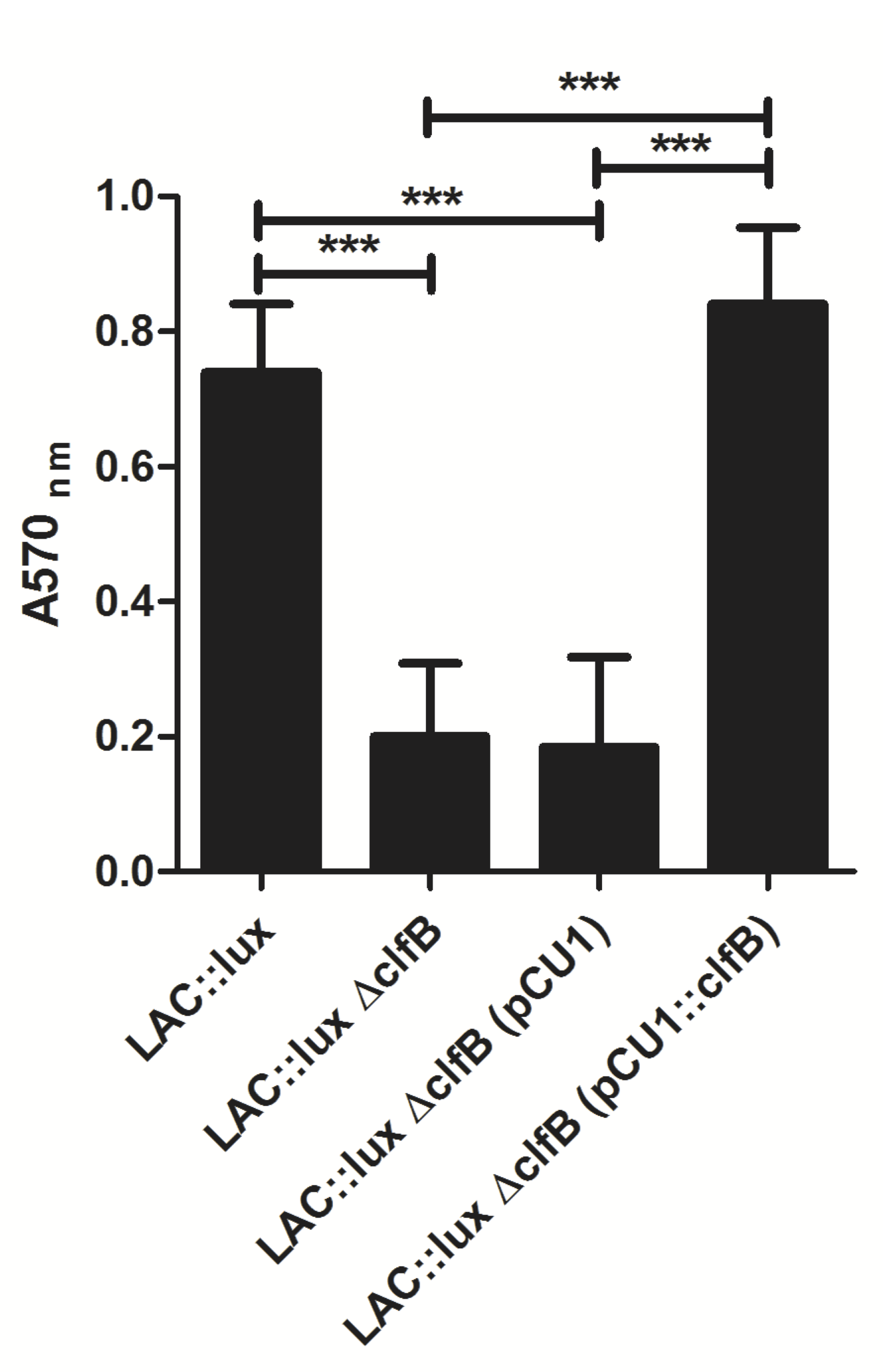

Supplement: S7 Fig — Microtiter plates were coated with GST-tagged loricrin loop 2 region (L2v, 0.3125 μg/ml). Adherence of S. aureus grown to exponential phase to immobilized L2v was assessed by staining with crystal violet and measuring absorbance at 570nm. Data pooled from 3 independent experiments. Error bars represent the standard deviation. One-way ANOVA with Tukeys post-test used to analyze differences between groups. *** P < 0.001. (TIF) [file ppat.1007713.s007.tif]

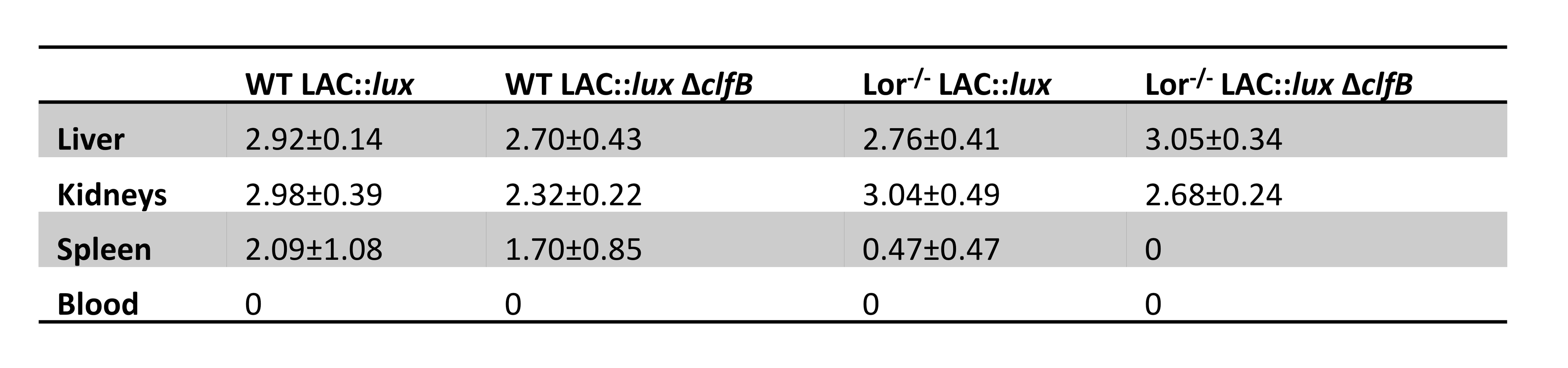

Supplement: S1 Table — Wild-type FVB (WT) and Lor-/- mice were infected subcutaneously with 2x10^7^ CFU S. aureus LAC::lux or LAC::lux ΔclfB and bacterial burden in the blood and peripheral organs assessed by viable counting at 24 hours post-infection. Results are expressed as Log10 CFU/mg. n = 3 per group. (TIF) [file ppat.1007713.s008.tif]

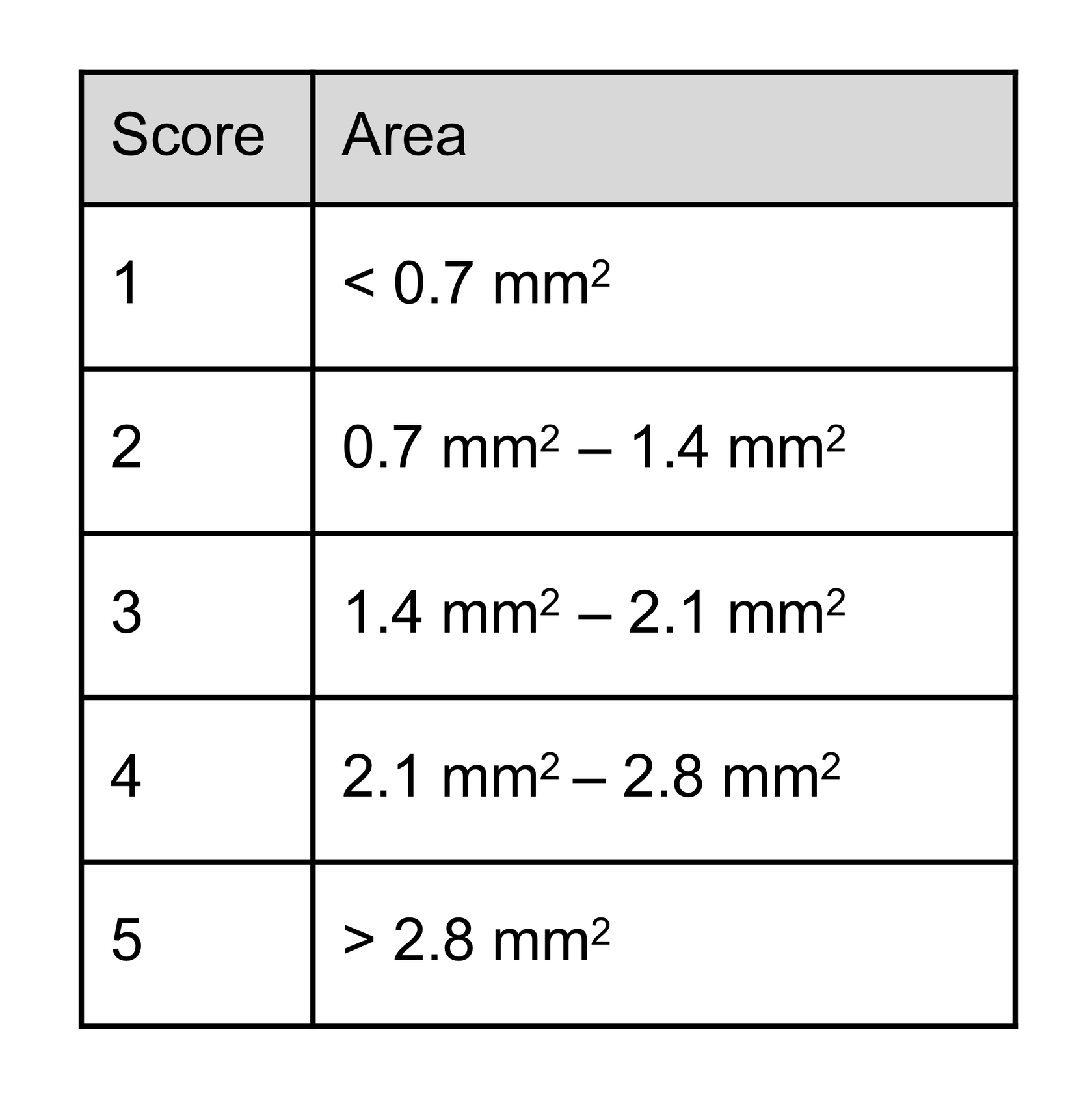

Supplement: S2 Table — (TIF) [file ppat.1007713.s009.tif]
